# microRNA profiling in the Weddell seal suggests novel regulatory mechanisms contributing to diving adaptation

**DOI:** 10.1186/s12864-020-6675-0

**Published:** 2020-04-15

**Authors:** Luca Penso-Dolfin, Wilfried Haerty, Allyson Hindle, Federica Di Palma

**Affiliations:** 1 Earlham Institute, Norwich Research Park, Colney Lane, Norwich, NR47UZ UK; 2 German Cancer Research Center (DKFZ), Im Neuenheimer Feld 280, 69120 Heidelberg, Germany; 3 Massachusetts General Hospital, 55 Fruit St, Boston, MA 02114 USA; 4 University of Nevada Las Vegas, 4505 S Maryland Pkwy, Las Vegas, NV 89154 USA

**Keywords:** microRNA, Hypoxia, Deep diving, Marine mammals, Adaptation, Evolution, Gene regulation

## Abstract

**Background:**

The Weddell Seal (*Leptonychotes weddelli*) represents a remarkable example of adaptation to diving among marine mammals. This species is capable of diving > 900 m deep and remaining underwater for more than 60 min. A number of key physiological specializations have been identified, including the low levels of aerobic, lipid-based metabolism under hypoxia, significant increase in oxygen storage in blood and muscle; high blood volume and extreme cardiovascular control. These adaptations have been linked to increased abundance of key proteins, suggesting an important, yet still understudied role for gene reprogramming.

In this study, we investigate the possibility that post-transcriptional gene regulation by microRNAs (miRNAs) has contributed to the adaptive evolution of diving capacities in the Weddell Seal.

**Results:**

Using small RNA data across 4 tissues (brain, heart, muscle and plasma), in 3 biological replicates, we generate the first miRNA annotation in this species, consisting of 559 high confidence, manually curated miRNA loci. Evolutionary analyses of miRNA gain and loss highlight a high number of Weddell seal specific miRNAs.

Four hundred sixteen miRNAs were differentially expressed (DE) among tissues, whereas 80 miRNAs were differentially expressed (DE) across all tissues between pups and adults and age differences for specific tissues were detected in 188 miRNAs. mRNA targets of these altered miRNAs identify possible protective mechanisms in individual tissues, particularly relevant to hypoxia tolerance, anti-apoptotic pathways, and nitric oxide signal transduction. Novel, lineage-specific miRNAs associated with developmental changes target genes with roles in angiogenesis and vasoregulatory signaling.

**Conclusions:**

Altogether, we provide an overview of miRNA composition and evolution in the Weddell seal, and the first insights into their possible role in the specialization to diving.

## Background

The Antarctic Weddell Seal (*Leptonychotes weddelli*) is a deep diving marine mammal, capable of pursuing prey to depths > 900 m and remaining underwater for more than 60 min [1, 2]. Due to their exceptional diving ability and accessibility on the fast ice during their breeding season, the Weddell seal is one of the best-studied divers in the world. The pinniped lineage recolonized the marine environment ~ 25 mya [3] and over this evolutionary time have become specialized to their aquatic habitat. These specializations encompass morphology and physiology; in particular, the extreme cardiovascular physiology of diving mammals is central to their capacity for long-duration diving. The well-developed dive response of marine mammals, including Weddell seals, is characterized by cardiovascular adjustments to lower heart rate and reduce peripheral blood flow during submergence. These adjustments depress tissue oxygen use by restricting its availability to peripheral vascular beds and conserving it for critical central tissues such as the brain and heart. Previous work has also highlighted several complementary traits that support breath-hold hunting in seals, for example: the preference for aerobic, lipid-based metabolism under hypoxia [4–6]; and extremely high oxygen stores in blood and muscle via enhanced haemoglobin and myoglobin [7–9].

Pinnipeds provide a fascinating model system in which to study the development of diving ability and hypoxia tolerance in mammals. Only adult seals are elite divers – unlike cetacean calves, pinniped pups are born on land. Development of the adult diving phenotype has been linked to changes in key proteins (e.g. respiratory pigments), tissue iron content and metabolic enzyme levels [5]. However, the details of the extent of tissue-specific maturation to refine local blood flow [10], metabolic control, and to combat negative effects of hypoxia exposure are still to be elucidated. Interestingly, pup physiology develops during weaning and throughout a post-weaning fast, including cardiac ontogeny to develop the fine-scale control of bradycardia observed in adults. This maturation can begin before pups first enter the water, which suggests an important role for gene reprogramming. The contribution of post-transcriptional gene regulation in the development of hypoxia tolerance and dive capacity has not been investigated.

MicroRNAs (miRNAs) are considered one of the key gene regulators in animals, conferring temporo-spatial precision in the regulation of gene expression. These short (~ 22 nt) non-coding RNAs are involved in fundamental processes such as embryonic development and tissue differentiation [11–14] and likely play important roles in seasonal and developmental transitions involving gene reprogramming. MiRNAs reduce translation by binding to the 3′ untranslated region (UTR) of complementary RNA, resulting in direct translational repression or mRNA degradation [15–17]. MiRNAs appear critical to the development of tissue-specific phenotypes and evolutionary adjustments in gene expression [18, 19]. For example, differential miRNA profiles in the highland yak compared to the lowland cow are enriched for hypoxia signaling pathways in the respiratory and cardiovascular systems – a key component of altitude adaptation [20]. These small non-coding RNAs also regulate seasonal phenotypic shifts, including metabolic depression that accompanies hibernation, anoxia tolerance and estivation in several vertebrates, although the exact miRNAs that may regulate these transitions appear species-specific [21, 22]. MiRNAs are also implicated in protection against environmental stresses such as hypoxia, extreme temperature and nutrient limitation in animals and plants [23–25].

In this study, we investigate a potential role for miRNAs in Weddell seal maturation, by post-transcriptional regulation of genes involved in development of the dive response and hypoxia tolerance. We provide the first comprehensive dataset of high quality, manually curated miRNA loci for this species, and use this dataset to investigate: 1) the patterns of differential expression of miRNAs across four tissues in pups and adults with a range of hypoxia sensitivity; 2) the putative mRNA targets for each miRNA; and 3) pathway analyses for the targets of differentially expressed miRNAs, focusing primarily on significant expression differences between pups and adults that could explain the development of diving capacity.

## Results

### Sequencing, alignment, and annotation

As an initial quality check, we mapped all adapter-trimmed reads against the *LepWed1.0* genome assembly, with no gaps or mismatches allowed. Approximately 70% of reads obtained from tissues aligned perfectly to the Weddell seal genome and only 43–55% of reads from plasma samples were perfectly aligned (Additional file 1: Fig. S1). These results illuminated a large portion of the read data that was unmapped to the Weddell seal genome (Additional file 1: Fig. S2). As several unaligned, putative miRNAs were highly expressed, we further identified reads that did not map to the Weddell seal genome, but were perfectly aligned to a miRNA hairpin sequence annotated in miRBase [26]. For example, the sequence TGAGATGAAGCACTGTAGCT was represented by more than half a million reads in each plasma sample (532,097-1,014,236 adapter-trimmed reads) with the only exception of *164393_1* (2095 reads). This sequence did not map to the Weddell seal genome, but was identified as miR-143-3p. The number of unmapped reads identified as miRBase annotated miRNAs ranged from 291,923 in a brain sample to 2,076,932 in a plasma sample. In 5 out of 6 plasma samples, these unmapped miRBase sequences accounted for > 1,000,000 reads. Thus, assembly incompleteness appears to reduce the percentage of small RNA reads that could be mapped to the Weddell seal genome, with the greatest impact on plasma samples.

In order to annotate high confidence miRNA loci, we manually curated the set of predictions provided by *miRCat2* [27] and *miRDeep2* [28]. This led to a final set of 559 loci (union of high confidence predictions for each tool; Additional file 2: Table S1; Additional file 1: Fig. S3). Among these, 329 corresponded to a *miRBase* annotated hairpin sequence, while the remaining 230 represent novel miRNAs.

We examined variation in the relative abundance of miRNA-5p and miRNA-3p across all samples and did not identify any clear examples of arm switching (i.e. changes of the most abundant miRNA strand).

### Differential miRNA expression among tissues

**Fig. 1:**
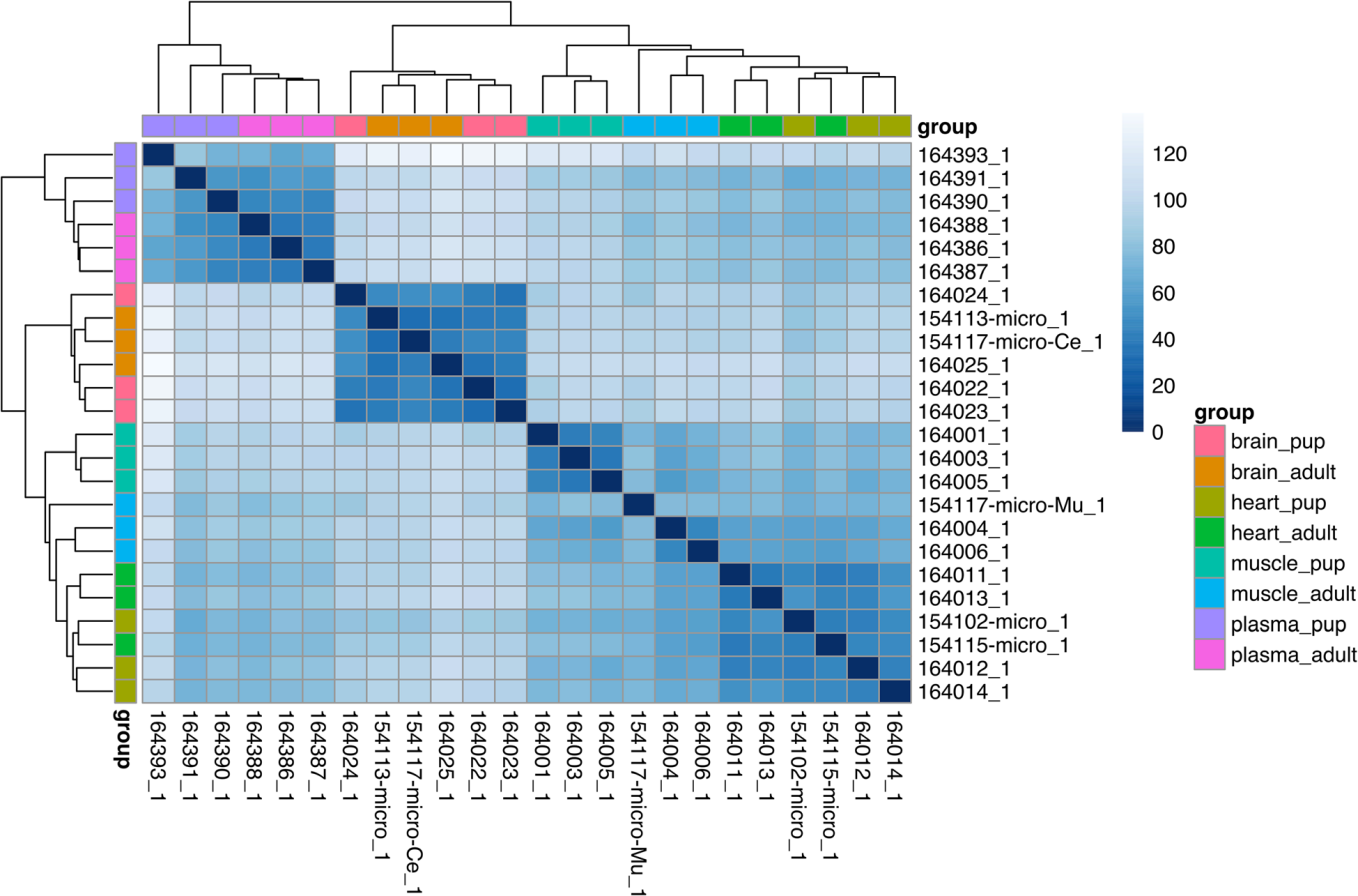
Heatmap of sample distances based on microRNA expression across 4 tissues. Gradient of blue corresponds to distance, while samples are color coded based on the tissue origin and developmental stage of the individual

**Fig. 2:**
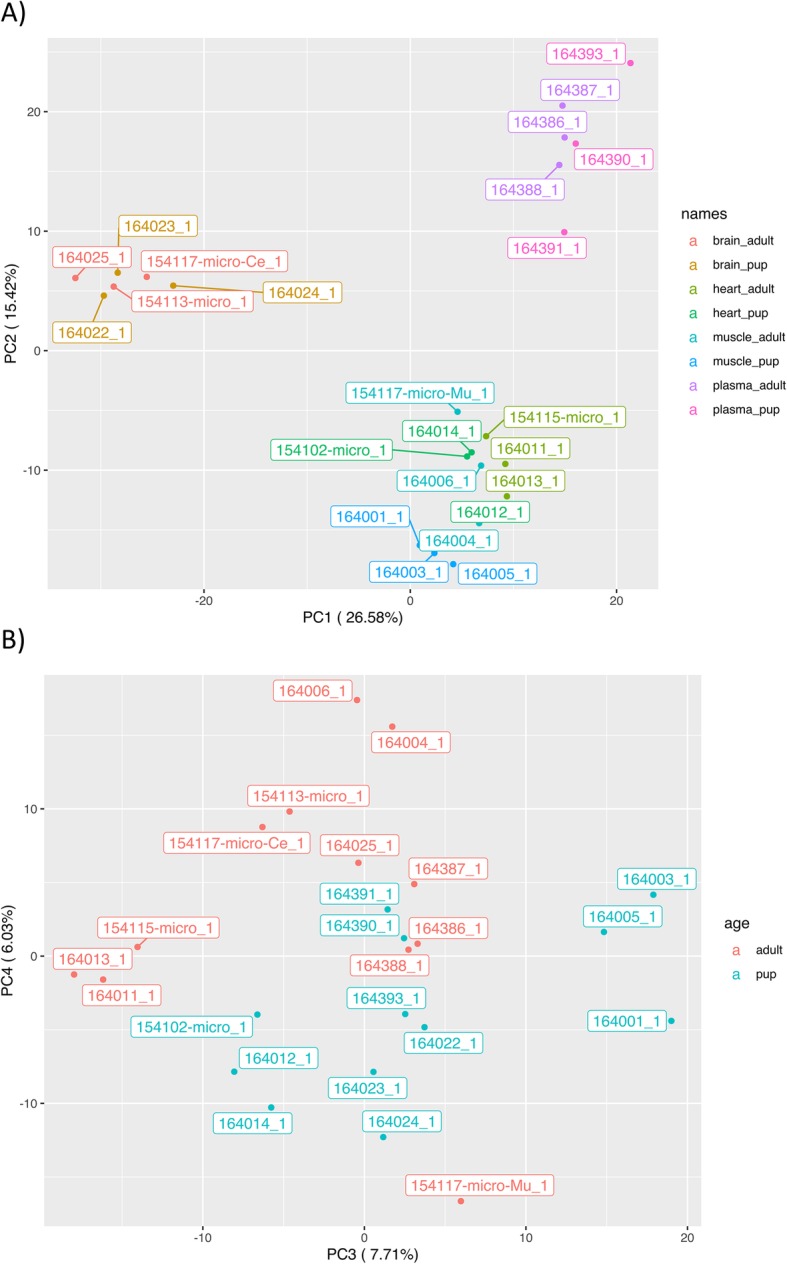
PCA plot based on microRNA expression across all 24 samples. **a** PCA plot of principal components 1 and 2. Colour labels are based on both the tissue origin and developmental stage of the individual. **b** PCA plot of principal components 3 and 4. Colour labels separate the two developmental stages (pup and adult)

**Fig. 3:**
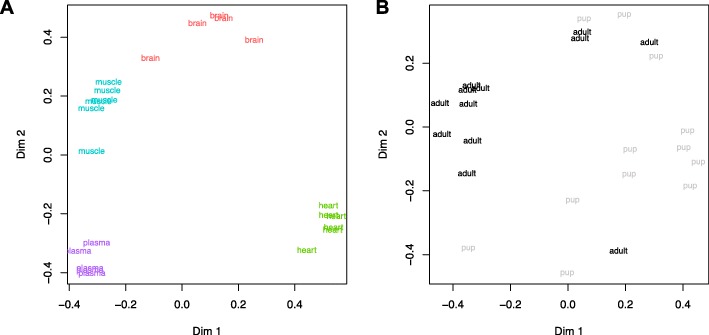
Random Forests plots classifying Weddell seal sample types based on a minimal set of microRNA abundance inputs. Panel **a** demonstrates clustering among tissue types (*n* = 6 samples per tissue) using 10 microRNA inputs. Panel **b** demonstrates clustering between developmental stages (*n* = 12 pup versus adult samples)

Four hundred sixteen miRNAs were differentially expressed (DE) in a single sample type compared to all others. Among these, 74 were DE in two tissues (490 significant changes in total, see Additional file 1: Fig. S4, S5). 50% of tissue-specific DE occurred in the brain, with 31% of significant results in plasma. A smaller set of loci were uniquely elevated or depressed in heart (8%) and muscle (11%; Table 1). These two contractile tissues were also the most similar to each other when expression data was viewed as a heatmap (Fig. 1), although all 4 tissue types were segregated by hierarchical clustering. Principal Component Analysis (PCA) clearly separated samples by tissue along PC1 and PC2 (Fig. 2a). As with the heatmap, PCA points to greater similarity between the contractile tissues, heart and muscle, relative to plasma and brain. Moreover, brain is clearly separated from the other tissues along the PC1 axis, with limited inter-individual variability, especially for PC2. Random forests analyses identified 6 miRNA inputs that were able to separate the four tissue types from each other with zero error, including one novel, unmapped Weddell seal miRNA novel-4-3p, whose expression is significantly upregulated in plasma and downregulated in muscle (Table 2; Fig. 3a). Four of these classifiers were upregulated in heart (miR-490-3p, miR-499-5p, miR-30e-5p and miR-30d-5p) and four were downregulated in plasma (miR-95-3p, miR-499-5p, miR-30a-5p and miR-30d-5p; Additional file 3: Table S5).

**Table 1:** Differential expression (DE) of miRNAs across tissues, with developmental stage, and in tissue-specific developmental comparisons

Comparison	downregulated miRNAs	upregulated miRNAs	Total DE
*Tissue analysis: relative to all other sample types*			
Brain	76	169	245
Heart	5	33	38
Muscle	6	50	56
Plasma	84	67	151
*Age analysis: adult relative to pup in all sample types combined*			
Adult vs. Pup	46	34	80
*Age x Tissue: adult relative to pup for each tissue*			
Brain	43	55	98
Heart	9	20	29
Muscle	67	26	93
Plasma	0	0	0

**Table 2:** MiRNAs that best discriminate Weddell seal tissue sampling locations or developmental stages by Random Forests analysis

MicroRNA Inputs	
*Tissue Selection*	
lwe-miR-499_loc1-5p	
novel_4-3p	
lwe-miR-490_loc1-3p	
lwe-miR-95_loc1-3p	
lwe-miR-30e_miRDeep2_loc1-5p	
lwe-miR-30d_miRDeep2_loc1-5p	
*Age Selection*	
lwe-miR-29a_loc1-3p	
lwe-miR-542_loc1-5p	

Note: Abundance (raw read counts) of the listed miRNA inputs were identified as those that best clustered the four tissues (brain, heart, skeletal muscle and plasma) or the two developmental groups (adult versus pup). Supervised classification in Random Forests analyses of the four tissue types had zero error, clustering of samples by developmental stage had 0.125 ± 0.068 out of bag error

To shed light on the biological role of miRNAs of interest, we mapped mRNA targets of DE miRNAs to significantly enriched gene pathways. miRNA downregulation within a tissue could infer higher local expression of target mRNAs. Therefore, the activity of pathways associated with upregulated miRNAs are potentially lowered, while pathways associated with downregulated miRNAs and their targets are potentially enhanced.

miR-499-5p was the most DE locus in the dataset, a Random Forests classifier, and has the highest expression in heart (Additional file 3: Table S5). mRNA targets of miR-499-5p, predicted to have lower expression in heart than other seal tissues include *Jade1*, a pro-apoptotic factor. miR-490-3p was also highly expressed in the heart, was a Random Forests classifier, and was among the five most significant DE changes in the dataset (Additional file 3: Table S6). miR-490-3p targets ion channels and transporters, including members of the KCN and SLC gene families (*Kcng3*, *Kcnj15*, *Kcne2*, *Kcne4*, *Kcnmb1*, *Kcnip3*, *Kcnk12*, *Slc1A3*, *Slc9a1*, *Slc9a2*), associated with pathway enrichments in potassium ion transmembrane transport (GO:0071804, GO:0071805). High relative expressions of both miRNAs are consistent with prior miRNA biomarker studies in heart [29, 30], and do not differ between pups and adults. Although KEGG disease pathways were excluded from the presented data, it is noteworthy that pathway analysis identified enrichment of three cardiac disease pathways (hsa05410: Hypertrophic cardiomyopathy, hsa05414: Dilated cardiomyopathy, hsa05412: Arrhythmogenic right ventricular cardiomyopathy) associated with mRNA targets of miRNAs that were significantly upregulated in hearts of Weddell seals.

The brain had more DE miRNAs than any other tissue, impacting the largest number of mRNA targets. In general, targets of DE miRNAs in the seal brain were enriched for neuronal and developmental processes (Additional file 3: Table S6). The largest pairwise fold change occurred in miR-488-3p (brain > plasma), which targets steroid hormone receptors including *Oxtr*, *Ghrhr*, and *Pgrmc2*. High relative expression in brain may downregulate the steroid hormone response pathway (GO:0048545).

miR-296-3p was among 6 downregulated miRNAs in seal swimming muscle. Enhanced pathways associated with targets of this miRNA include those expected for skeletal muscle, related to ryanodine receptor redox state regulation, calcium handling and the sarcoplasmic reticulum (GO:0060314, GO:0033017, GO:0016529, GO:1901019, GO:0050848), as well as response to extracellular acidic pH (*Rab11fip5*, *Rab11b*, *Impact*, *Asic1*, *Asic2*, GO:0010447). We also identified elevated miR-206-3p in muscle, which [31] has been identified as a *MyomiR*, with expression restricted to this tissue. In contrast to heart, miR-490-3p was lowest in muscle, pointing to an enrichment of target ion channels and potassium transport. Both strands of miR-10 are upregulated in muscle. Although the 5p strand is most abundant (average 5p read counts: 2,233,312 in pups and 1,634,627 in adults; average 3p read counts: 413.6 in pups, 212 in adults) the 3p has large tissue-specific fold changes (23-62X) compared to other tissues, downregulating predicted targets related to the regulation of blood circulation (GO:1903522), including *Bves*, *Casq2*, and *Nos1*.

Of the Random Forest classifiers DE in plasma (Table 2), miR-95-3p had the largest sample-specific downregulation (20-53X lower than heart, muscle, and brain; Additional file 3: Tables S3, S4, S5). miR-95-3p targets LDL receptor related protein (*Lrp1*) as well as the beta-subunit of guanylyl cyclase (*Gucy1b1*). miR-339-3p, also downregulated in plasma compared to other tissues, targets several key genes in hypoxia sensing (GO:0036293, GO:0070482, GO:0001666) including *Epas1*, *Vhl*, *Hif1an*, and *Cygb*, but does not change with age (Additional file 1: Fig. S6). Several miRNAs elevated in Weddell seal plasma versus other tissues (miR-18a-5p, miR-221-3p, and miR-34-5p) target pathways regulating blood vessel and vascular remodeling (e.g. GO:0001974), with miR-34-5p specifically targeting *Epas1* and angiotensin (*Agt*).

### Differential miRNA expression with development

Eighty miRNAs were DE between adults and pups across all tissues (Table 1). One hundred eighty-eight miRNAs were DE in tissue-specific developmental comparisons (27 of these miRNAs were significantly up or downregulated in more than one comparison, see Additional file 1: Fig. S7). Heatmap visualization also separates pups from adults within each tissue (Fig. 1). An exception is the miRNA profile of adult skeletal muscle, which clusters more closely with heart samples than with muscle from pups. PC3 and PC4 provide the best developmental clustering (Fig. 2b) but explain only 13.7% of variation. Supervised clustering by Random Forests (Fig. 3) identifies 2 miRNA inputs (miR-29a-3p, miR-542-5p) that separate the samples by developmental stage for all tissues combined (Table 3; Fig. 3b). miR-29a-3p is significantly upregulated in brain, heart and muscle of adults, whereas miR-542-5p is downregulated in adult brain and muscle (Additional file 3: Table S7). Brain and skeletal muscle miRNAs had large developmental differences among the four sample types investigated (Table 1). Only 13% of significant adult-pup differences occurred in the heart and there were no developmental differences in plasma miRNAs (Table 1).

**Table 3:** The number of unique mRNA targets of differentially expressed miRNAs in four target tissues, with developmental stage, and in tissue-specific developmental comparisons

Targets of DE miRNAs	Targets of downregulated miRNAs	Targets of upregulated miRNAs
*Tissue analysis: relative to all other sample types*		
Brain	7450	10,172
Heart	580	3962
Muscle	1643	4668
Plasma	7426	7249
*Age analysis: adult relative to pup in all sample types combined*		
Adult vs. Pup	4125	4572
*Age x Tissue: adult relative to pup for each tissue*		
Brain	4560	9111
Heart	920	2830
Muscle	5900	4144
Plasma	0	0

Predicted targets of miR-29a-3p that would be reduced from a terrestrial pup to a diving adult include components of the mitochondrial electron transport system (*Cox4i1*, *Cox10*, *Ndufs7*, *Ndor1*, *Sdhaf2*), genes related to iron regulation (*Tfrc*, *Ireb2*) and negative regulators of hypoxia signaling (*Hif3a*, *Vhl*). Conversely, higher miR-542-5p in pups is predicted to enhance the abundance of mRNAs that are likely relevant in the adult phenotype. Genes of interest include the mitochondrial citrate transporter *Slc25a1*, as well as genes known to regulate hematopoiesis (*Cd44*), anaerobic glycolysis (*Ldha*), and obesity/lipolysis (*Plin1*).

### High number of lineage specific miRNA orthogroups

**Fig. 4:**
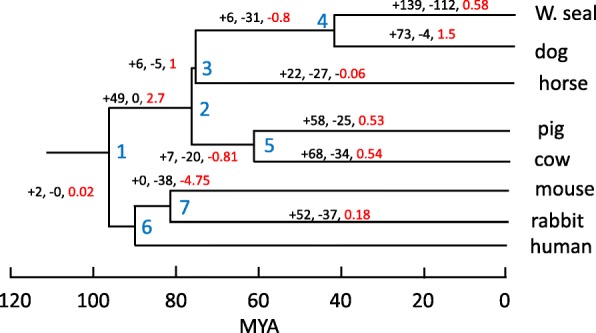
The gain and loss of mammalian miRNA orthogroups in 8 mammals, inferred by Dollo parsimony and synteny analyses. Number of gained (+) and lost (−) orthogroups are listed for each branch of the tree (black). Red numbers represent the branch-specific net gain rate of orthogroups per million year

Recent studies have highlighted the high rate of novel miRNA gains in mammals [32, 33]. We identified 874 miRNA sequence clusters in a dataset that included Weddell seal, cow, dog, horse, pig, rabbit, mouse, and human (see Methods). These were considered miRNA orthogroups and were evaluated to infer the evolutionary patterns of gain and loss across the phylogenetic tree (Fig. 4). High net gain rates in both the dog and the Weddell Seal lineages suggest dynamic miRNA evolution. For the seal, we also observe a high number of lineage specific losses. This may reflect purifying selection on young, selectively neutral miRNAs, however this result might be biased by differences in assembly completeness between the dog [34] and the Weddell seal genome references.

**Fig. 5:**
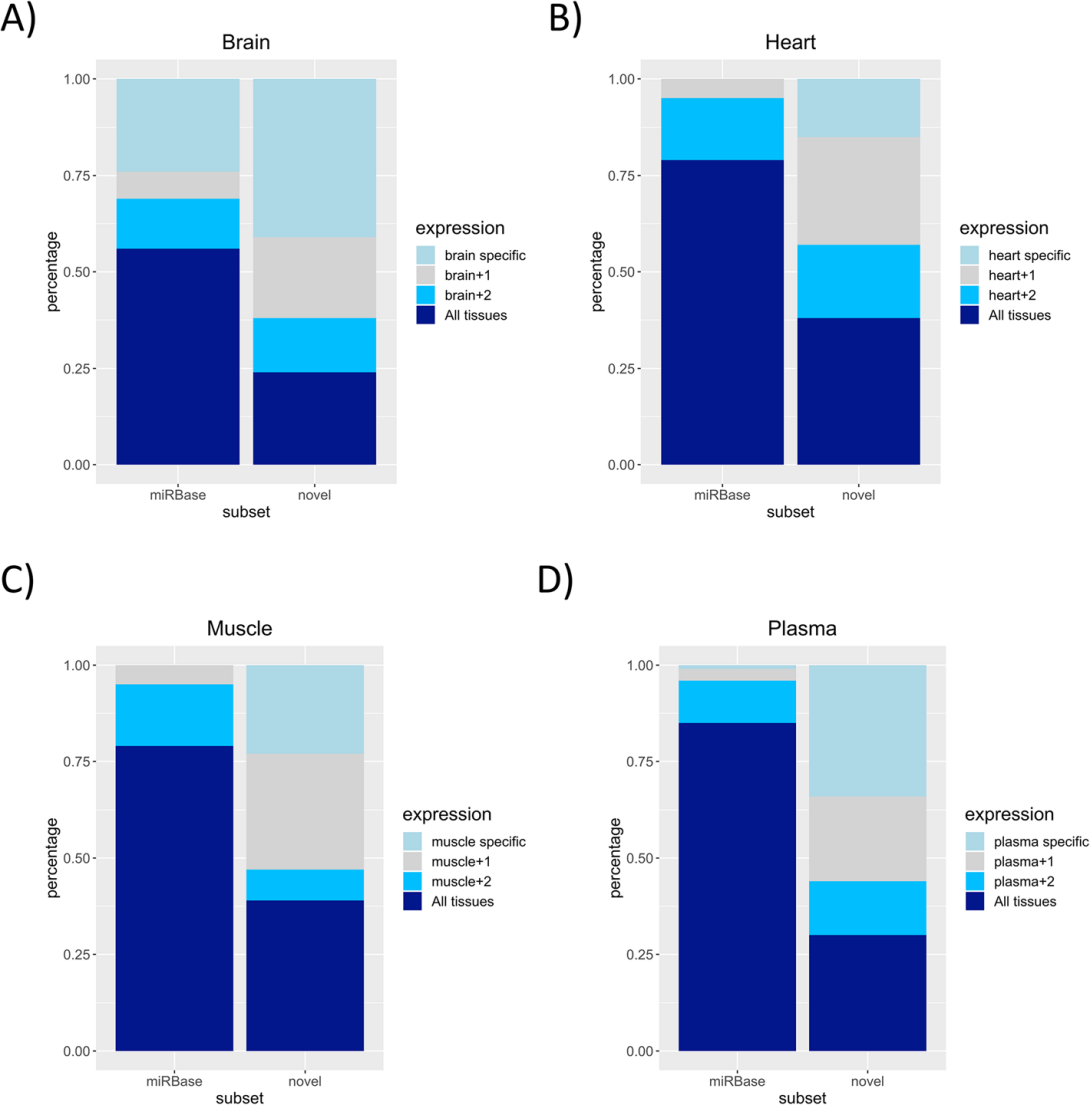
Expression patterns of novel and miRBase orthogroups in 4 sample types. The “miRBase” category contains orthogroups that contain annotated miRNAs, whereas orthogroups with unannotated members are classified as “Novel”. Orthogroups with mixed composition (containing both novel and miRBase genes) were classified as “miRBase”. Colouring denotes the percent of miRNA families expressed in each tissue that are tissue-specific, expressed in the tissue of interest and one (+ 1) or two (+ 2) other sample types, or are expressed in all tissues. **a** brain; **b** heart; **c** muscle; **d** plasma

Novel Weddell seal miRNAs are more likely to be tissue-specific, as we observed a significantly higher proportion of tissue-specific miRNA families in the novel set compared to the miRBase set (z test, brain: *p* = 0.0001; heart: *p* < 10^− 4^; muscle: *p* < 10^− 4^; plasma: *p* < 10^− 4^; Fig. 5). This aligns with previous findings, which suggest that young miRNAs are initially expressed in a single or few tissues, then become more broadly expressed later in their evolutionary history [32].

Predicted mRNA targets of novel Weddell seal miRNAs have a functional overlap with targets of annotated miRNAs in this dataset, as evidenced by similarities in pathway enrichments for tissue-specific up- and down-regulated miRNA targets, regardless of whether miRNAs were previously annotated or novel in Weddell seals. For example, VEGF signaling was targeted by both novel and annotated miRNAs in the seal heart, highlighting the importance of vascular development in cardiac tissue. We specifically investigated pathways identified from novel Weddell seal miRNAs, to evaluate the capability for species-specific gene regulation and manifestation of phenotype.

**Fig. 6:**
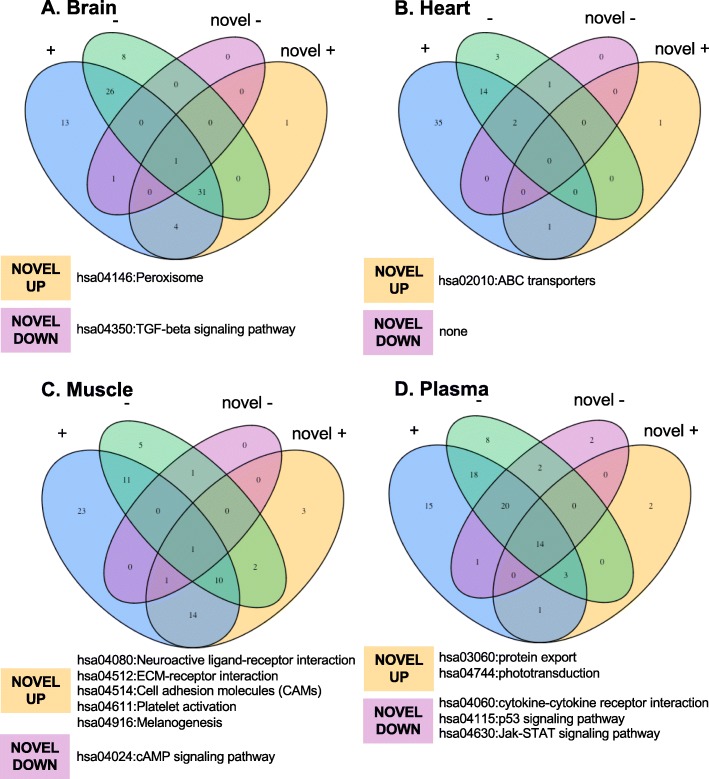
Venn diagrams demonstrate overlap between enriched pathways of mRNA targets predicted to vary across 4 sample types in Weddell seals. Enriched Kegg pathways were identified based on miRNAs significantly up (+) or down (−) regulated in each tissue compared to all others and categorized as pathways related to previously annotated miRNAs versus novel, unannotated miRNAs identified in the Weddell seal (novel +, novel -). For each direction of regulation, the identities of enriched pathways targeted by novel miRNAs that were not detected from annotated miRNAs with the same direction of differential expression are listed for each tissue. **a** brain; **b** heart; **c** muscle; **d** plasma

We compared pathways targeted by DE novel versus miRBase-annotated miRNAs, identifying unique pathway targets of novel miRNAs in each tissue. Thirty-eight pathways were enriched (*p* < 0.05, see Methods) for the targets of miRNAs that were highest in the brain and 31 pathways were enriched for targets of muscle-elevated miRNAs, which are primarily signaling pathways (Additional file 3: Tables S9, S10). Conversely, only two pathways in each tissue were associated with tissue-specific downregulated miRNAs. Only a limited selection of pathways were enriched by novel miRNAs that were not similarly identified from annotated miRNAs. Several of these pathways are associated with lipid metabolism (Peroxisome in brain, ABC transporters in heart) and inflammatory signaling (Cytokine-cytokine receptor and Jak-stat signaling in plasma, CAMs in skeletal muscle), both important elements of the Weddell seal phenotype (Fig. 6).

Specific novel miRNAs also indicate potential to control key biochemical and physiological features of the Weddell seal. Novel-15-3p, highly expressed in the Weddell seal heart, is associated with cardiomyopathy through its target dystrophin. Novel-10-3p is upregulated in plasma and is predicted to target 610 mRNAs, including those with vasoactive (*Nos1*, *Nos1ap*), iron modulating (*Hmox1*, *Hfe*), and lipid metabolic functions (*Lep*).

### Targets of differentially expressed miRNAs control physiological changes associated with elite diving

Within each tissue, age differences in gene expression, driven by miRNA regulation detected in this dataset likely support established developmental patterns of mammals generally [35, 36]. Beyond this, we examined the 220 cases of DE between adult and pup in either the brain, heart, or muscle specifically, which may regulate the development of the diving phenotype (Additional file 3: Table S7). No age-specific differences were identified in plasma miRNAs (Table 1).

Several pathway enrichments and specific predicted target mRNAs related to hypoxia signaling were detected in this dataset (Additional file 3: Table S8). The main developmental signal linked to hypoxia responses was the expression of miR-424-3p, which is significantly reduced in the heart overall, and additionally downregulated in the adult seal compared to pup. *Egln3* is a prolyl hydroxylase involved in HIF1α degradation under normoxic conditions and is predicted to be targeted by miR-424-3p. *Egln3* also hydroxylates pyruvate kinase (PKM) in hypoxia, restricting glycolysis.

**Fig. 7:**
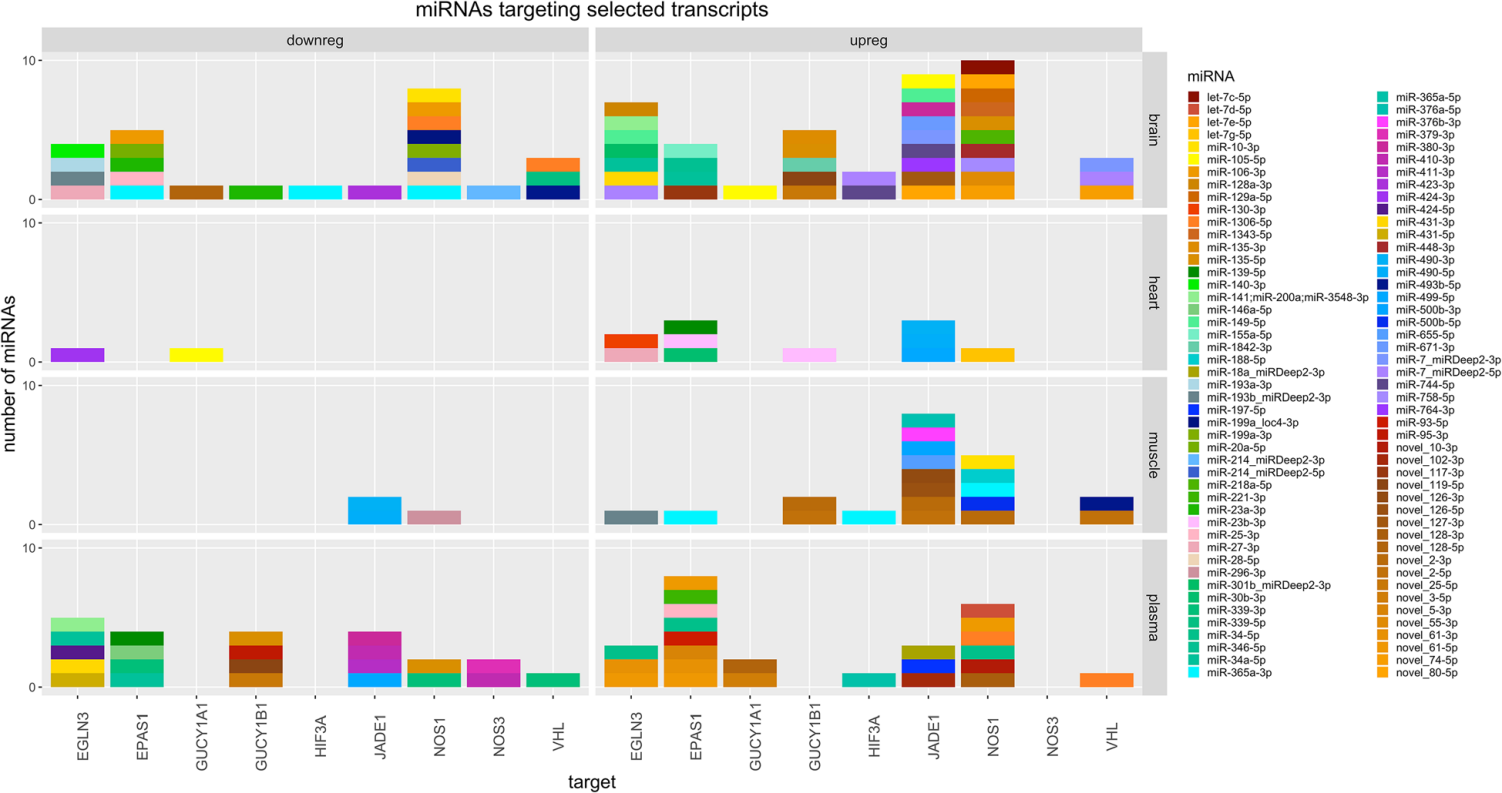
Selected targets of miRNAs DE between tissues. Rows correspond to tissues, columns to the direction of the expression change. Groups of miRNAs targeting the same gene in the same DE group are represented by stacked columns, with a height proportional to the number of different miRNAs. Each miRNA is assigned a specific color label (see legend)

Each tissue type revealed DE miRNAs that target nitric oxide (NO) signaling (Fig. 7). Differentially upregulated miRNAs target the beta-1 subunit of guanylyl cyclase (*Gucy1b1*), which would decrease local expression of the heterodimeric enzyme in the brain, heart and muscle overall, whereas miRNAs targeting *Gucy1b1* were downregulated in plasma (Fig. 7). Within the brain, an additional upregulation of novel-25-5p and novel-119-5p was detected in adult seals, which also target *Gucy1b1* (Additional file 3: Table S7). Guanylyl cyclase is a major target of NO and has previously been shown to be reduced in seal tissues relative to terrestrial mammals [10]. The alpha-1 subunit (*Gucy1a1*) is also targeted by several DE miRNAs, including novel-128-5p (Fig. 7). Novel-128-5p is highly expressed in plasma and is also upregulated overall with age (Additional file 3: Tables S1, S6). In addition to miRNAs that may regulate endothelial NO signal transduction via guanylyl cyclase, a number of miRNAs in this dataset target the NO synthase (*Nos1*) directly (Fig. 7, Additional file 3: Table S7), with age-related DE pointing to increased *Nos1* mRNA expression in adult muscle (miR-377-5p, miR-214-5p, -miR-543-3p) but decreased expression in adult brain (miR34-5p, miR-1306-5p).

Contrary to central tissues such as the heart and brain, muscles experience reduced and potentially episodic blood flow during diving [37, 38]. Targets of age-increased miR-218a-3p are enriched for ubiquitin/proteasome pathways, suggesting a less robust response to protein damage in adults, as well as reduced apoptosis signaling (miR-29c-3p, miR-129a-3p).

Of 137 novel miRNA loci (133 orthogroups) identified in gain and loss evolution analyses as specific to the Weddell Seal lineage, 9 mature miRNAs are DE between adults and pups in the brain, 4 are DE with age in the heart, and 6 are DE with age in muscle (Additional file 3: Table S7). Four of the novel miRNAs upregulated in the adult brain and 2 that are upregulated in the adult heart are predicted to target *Vash1*, an angiogenesis inhibitor selective to endothelial cells. Three miRNAs are upregulated in both the heart and skeletal muscle of adult seals and the predicted mRNA targets of these three miRNAs include *Ednra*, the receptor for endothelin 1 (a potent vasoconstrictor), as well as several members of the lipid transporter ABCA gene family (*Abca8*, *Abca10*, *Abca12*).

## Discussion

In this study, we provide the first annotation of the Weddell seal miRNome, by manually curating the computational predictions of *MiRCat2* and *MiRDeep2*. We identified 559 high confidence miRNA loci; 146 of these represent novel miRNAs absent from *miRBase*. Evolutionary and comparative analyses highlight high lineage-specific miRNA gains in the Weddell Seal, compared to other mammals. Expression of these novel miRNAs tends to be tissue-specific, relative to *miRbase*-annotated miRNAs that are more widely expressed. mRNA targets were assigned to DE miRNAs using a highly conservative approach. As a result, differential miRNA expression by tissue identifies known miRNA biomarkers that have been linked to tissue-specific mRNAs and function, as well as previously undocumented mRNA targets of known and novel miRNAs that are likely associated with seal-specific physiology.

### Protective mechanisms relevant to diving in each tissue

Differential expression of cardiac miRNAs are the most limited in the dataset but represent some of the largest magnitude changes, which highlight miRNAs related to ischemic stress. In cardiomyocytes, miR-499-5p is protective against myocardial infarction due to anti-apoptotic action [39]. In particular, the pro-apoptotic *Jade1* target is predicted to be reduced in the seal heart due to high miR-499-5p, suggesting a protective mechanism against cardiac ischemia [40]. Circulating miR-499 is considered a biomarker of myocardial infarction [30] and this miRNA was not elevated in plasma nor affected by age, suggesting no underlying heart damage in seals despite the intense cardiac regulation involved in the dive response, and the reported potential for cardiac dysregulation in the conflict between the bradycardia associated with diving and the tachycardia associated with exercise [41]. miR-499 is also important in ischemic postconditioning following cardiac challenge by ischemia-reperfusion [42]. miR-30d-5p oxer-expression limits ischemic injury and has demonstrated experimental ischemic protection in other tissues [43]. miR-490-3p is anti-atherosclerotic in human coronary artery smooth muscle cells [44].

Contrary to a central tissue such as the heart, skeletal muscles draw down local oxygen stores during diving [37, 38]. Exercise requirements of swimming muscle such as the *Longissimus dorsi* may be high as seals hunt below the ice, increasing reliance on anaerobic ATP production. *Asic3*, an acid channel and sensor for lactic acidosis [45] is predicted to be elevated in Weddell seal muscles via downregulation of miR-206-3p. Activation of *Asic3* contributes to neurally controlled local vasoconstriction, which would facilitate high peripheral vascular resistance and maintenance of the dive response despite hypoxia. Protective elements such as intrinsically high antioxidants have previously been identified in seal muscles [46, 47], and muscle miRNAs that are upregulated with age suggest dampening of other pathways that promote cell stress (ubiquitin/proteasome pathways, apoptosis signaling).

Brain and plasma have the most DE among miRNAs, likely to due to the high heterogeneity of tissue types found in the brain, and the contribution of many tissues releasing miRNAs into the venous circulation. Deep diving Antarctic phocids, including Weddell seals, demonstrate hypercortisolemia, however extremely high cortisol binding capabilities produce low free circulating cortisol [48]. These physiological findings are speculated to be a component of protection for the deep diving brain, possibly preventing high pressure nervous syndrome. While the brain is expected to be an enriched target for many hormone signaling pathways, it is potentially relevant that the largest DE miRNA in the brain (miR-488-3p) also targets steroid hormone receptors.

### Protective mechanisms induced with age and development

Seal pups are novice divers, limited biochemically and physiologically from elite diving [49]. One important component of seal pup maturation is the development of body oxygen stores. Adults maintain aerobic metabolism during routine diving by large oxygen storage abilities of the blood and muscle [50–52]. Rapid synthesis of respiratory pigments (hemoglobin and myoglobin) requires iron, and seal pups do not appear to meet iron needs through intake during the nursing period, requiring mobilization of internal irons reserves [53, 54]. Both *Tfrc* and *Ireb2* respond to low cellular iron levels [55, 56], and their expression could be diminished by miRNAs during development. This may be a regulating factor that minimizes the consequences of high iron turnover and depletion through respiratory pigment biosynthesis.

Increased skeletal muscle LDH activities to exploit anaerobic metabolism in extended diving have been shown in developing seals [57], however the ability to detect statistical differences between pups and adults appears to depend on the species-specific maturity of pups at birth, or pup age at the time of sampling [58]. No significant developmental difference in LDH enzyme activity was detected in Weddell seal *Longissimus dorsi* when 3–5 week old pups were compared to adults [59], however it is possible that this difference exists for younger pups (~ 1 week old in this study). Downregulation of transcripts encoding proteins of the electron transport chain in skeletal muscle with age, predicted from this study, are however consistent with significantly reduced mitochondrial volume densities in Weddell seal adults compared to pups from the same study [59].

### Regulation of perfusion

Tissue-specific control of perfusion is also important to cardiovascular regulation during diving [38, 60–63]. In terrestrial animals, the vasodilator nitric oxide (NO) improves perfusion and oxygen delivery in hypoxia. In diving seals, a NO response could be detrimental, as high peripheral vascular resistance is required to maintain central arterial pressure with submergence bradycardia. Indeed, downregulation of the nitric oxide-guanylyl cyclase-cGMP signal transduction pathway is limited in Weddell seals [10], which may facilitate central nervous system control of the dive response, but also reduce production of harmful peroxynitrites in the presence of reactive oxygen species. The number of DE miRNAs targeting this pathway, in particular novel miRNAs, strongly suggest that lineage-specific miRNA regulation contributes to control of local vasoregulators in the Weddell seal, and perhaps the evolution of diving capabilities. The most significant mRNA target of the pathway in this dataset is NO synthase 1 (*Nos1*). Although it is described as the neuronal form, *Nos1* has wide expression across tissues and in additional to functions in the brain, plays a role in systemic control of vascular smooth muscle tone [64] and myocardial function. Furthermore, *Nos1* is linked to calcium handling, as a regulator [65] and in its activation [66], and calcium signaling is enriched for mRNA targets of DE miRNAs in all tissues.

### Regulation of hypoxia tolerance

Despite extensive physiological adaptations to increase body oxygen stores and behavioral selection for aerobic diving, Weddell seals can experience dramatic, global hypoxemia during submergence [63]. This dataset detected a suite of miRNAs that target elements of HIF1α signaling, but only few miRNAs targeted HIF1α itself. For example, miR-424-3p, which regulates HIF1α through interactions with proteins in the ubiquitin-ligase system, as well as promotes angiogenesis in vitro in mice, is significantly reduced in heart compared to other tissues and further downregulated in the adult heart compared to the pup. Although low at baseline in Weddell seals, this miRNA is hypoxia-responsive in human endothelial cells [67]. Further, miR-29a-3p expression is increased in heart and muscle of adults versus pups, which is predicted to decrease expression of *Hif3a* and *Vhl*, both negative regulators of hypoxia signal transduction by HIF1α. Similar miRNA targeting of hypoxia signaling pathways was highlighted as an adaptation to high altitude in the yak, however a distinct set of miRNAs of interest were identified [20].

### Novel microRNAs as evolutionary avenues to regulate lineage-specific function

Our results confirmed previous observations of highly dynamic miRNA evolution, and additionally highlighted high net gain rates in both the dog and the Weddell Seal lineages. Novel, Weddell seal-specific miRNAs are evolutionarily young, and may provide key insights into the evolution of the diving phenotype. Similar pathway targets were identified in this hypoxia tolerant diver compared to the high-altitude, hypoxia-tolerant yak; however, they appear to be under the control of different sets of miRNAs in the 2 species. This strongly suggests that lineage-specific regulation is crucial to developing phenotypic adaptation in individual species. Additionally, novel miRNAs highlight mRNA targets that were previously overlooked. ~ 50% of novel miRNAs which are increased in the adult brain and heart target *Vash1*, an angiogenesis inhibitor selective to endothelial cells. These data could be interpreted to underscore the importance of maintaining vascularization and perfusion in two critical tissues. Three miRNAs are upregulated in both the heart and skeletal muscle of adult seals, implicating them as potential targets for future studies related to contractile tissue function, particularly as they share the potential to regulate the expression of the receptor for endothelin 1.

### Limitations and considerations

This analysis relied on developmental differences between pup and adult Weddell seals to highlight biological significance. Key differences among the tissues are informative towards the biology, but are difficult to interpret in the context of species-specific adaptations without comparison to background(s) of tissue-specific miRNAomes in other species. As with any genome study, this analysis is limited by the quality and completeness of available sequences. miRNA sequences and expression levels were generated specifically for this study, and subject to strict quality controls. The detection of miRNA targets relied on the 3′ UTR multiple alignments available in the *TargetScan7* database [68]. While our results were not experimentally confirmed, our approach was highly conservative, requiring miRNA-mRNA interactions to be independently predicted by three different algorithms. Moreover, miRNAs are capable of propagating regulatory signals by affecting the expression of other miRNAs [69], which were not considered. Functional confirmation of these interactions and their biological roles in the Weddell seal would be important future work towards understanding the molecular basis of the diving phenotype.

It is also important to acknowledge details of the sample collection which may impact interpretation of the results. In particular, this study included a sampling difference between tissues and plasma. Whereas plasma samples were collected from healthy animals in a brief handling event, heart, brain and muscle samples were collected at necropsy. All samples were collected rapidly from carcasses prior to freezing, however the smaller bodied pup carcasses could freeze quickly, whereas adults could be sampled within hours of death, leading to possible differences in miRNA degradation between age classes. This difference would not occur in plasma samples, which were processed similarly in both pups and adults, providing a potential explanation for the lack of age differences in plasma. However, the similar proportion of upregulated and down regulated adult-pup differences for the three tissues sampled at necropsy argues against a confounding factor associated simply with degradation differences in adult tissues. More likely to have impacted adult-pup miRNA differential expression in plasma versus the other tissues is a difference in pup age between the two sampling strategies. Plasma was collected during a brief handling event from weaned pups, so as to avoid disrupting mother-pup interactions prior to weaning. Pups that perish, by contrast, tend to be much younger, and within the first week of life. The lack of adult-pup differences in plasma samples within this dataset may reflect the additional maturation occurring in pups through the lactation period and past weaning.

## Conclusions

In this study, we take the first steps towards answering the question of whether post-transcriptional regulation by miRNAs has played a role in the evolution of diving capacities in the Weddell Seal. To address this, we have performed differential expression analyses, and identified significant differences in miRNA expression among tissues and between developmental stages. Targets of differentially expressed miRNAs are enriched for GO categories relevant to Weddell seal physiology, suggesting that miRNAs have indeed contributed to the evolution of extreme diving capabilities, and providing a new set of mechanistic hypotheses for future follow-ups. We also present a thorough miRNA annotation of the Weddell seal genome, representing a valuable resource for future biomedical and evolutionary studies. Elite diving seals have long been considered a potential model system in which to address human therapies associated with hypoxia and hyperlipidemia, however these links have been tentative due to the lack of available genomic resources.

Recent studies in vertebrates have highlighted expression divergence between vertebrate lineages, and the crucial role of gene regulation in the evolution of adaptive phenotypes [70–76]. The short sequence of a miRNA can quickly be gained or lost in an evolutionary lineage, possibly leading to the rapid acquisition of novel regulatory patterns. This study provides further indication of their evolutionary potential. It is yet another step towards a wider description of miRNA evolutionary dynamics in mammals, which can further clarify the contribution of gene regulatory mechanisms in the evolution of adaptive traits.

## Methods

### Animals and sample collection

Samples were collected from wild Weddell seal adults and pups near McMurdo Station, Antarctica as part of a larger study [10]. Tissue samples (heart right ventricle, *longissimus dorsi* swimming muscle, cerebral cortex) were collected at necropsy from animals found dead of natural causes on the sea ice (pups were ~ 1–7 days of age). Necropsies were performed on fresh carcasses, before tissues began to freeze. Tissues were rinsed in cold PBS, blotted dry, then either snap frozen in liquid nitrogen or preserved in an RNA stabilization solution (RNAlater, ThermoScientific AM7021). Venous blood samples were collected into EDTA-vacutainers from healthy adults and weaned pups (~ 35–45 days of age) during a brief handling period as previously described [10]. Plasma was isolated from whole blood by centrifugation (3000 *g* for 15 min) within an hour of collection in Antarctica. Animal demographic information is provided in additional file 3, Table S2. All animal handling and sample export was conducted under appropriate scientific authorizations (National Marine Fisheries Service #19439, #18662, ACA #2106–005) and approved by the Massachusetts General Hospital IACUC.

### Library preparation and sequencing

We extracted RNA from a total of 24 samples (*n* = 3 adult and *n* = 3 pup samples from each of the 3 tissues plus plasma). Total tissue RNA was isolated from tissues using the *miRNeasy Mini* kit (Qiagen #74104), then enriched for small RNAs using the *RNeasy MinElute Cleanup* kit (Qiagen #74204). miRNAs were extracted from plasma (*n* = 3 adults, *n* = 3 weaned pups) using a miRNeasy Serum/Plasma kit (Qiagen #217184). Enriched fractions were sent to *Macrogen* (South Korea), for small RNA library preparation and sequencing. Libraries were constructed for 24 high quality samples (minimum 100 ng RNA). All 24 libraries were barcoded, then pooled for sequencing using *Illumina HiSeq 2500*, with 10% *PhiX* sequencing control (Illumina FC-110-1003) included to increase sequence diversity. Resulting read data from small RNAs were validated using FASTQC [77].

### microRNA annotation

We annotated miRNA loci within the Weddell seal genome based on the combined set of small RNA libraries across all tissues and developmental stages, using both *miRCat2* [27] and *miRDeep2* [28] (Additional file 1: Fig. S3). The genomic coordinates predicted by *miRCat2* and *MiRDeep2* were then merged using *Bedtools merge* [78] to generate a non-overlapping set of loci. We then aligned small RNA reads of each library to our predicted hairpins. Following the methodology described in [33], predictions were then filtered based on the following criteria: evidence of both miR-3p and miR-5p expression in small RNA reads; alignments against the predicted loci with no gaps or mismatches allowed (Additional file 3: Tables S3-S4); miR-like hairpin secondary structure (as predicted using the *Vienna-RNA* package) [79]; and a minimum of 10 reads mapping perfectly to the predicted locus. This led to the generation of the final annotation, consisting of 559 high confidence miRNA loci.

### Clustering analyses

Dendrograms based on hierarchical clustering were generated in *RStudio* [80] version 1.2.1335 and plotted as a heatmap using the *R* package *DeSeq2* [81]. Strand specific miRNA expression data were used as input, after transformation with the *varianceStabilizingTransformation* function. Principal Components (PC) Analyses was performed in *RStudio* version 1.2.1335 using the *prcomp* function. Similar to the clustering analysis, transformed strand specific miRNA expression data was used as input. Supervised clustering was conducted using Random Forests [82] in variable selection mode to identify the minimum miRNA inputs required to best separate the samples by either tissue type (combining pup and adult data) or developmental stage (combining data from all tissues). Analysis was implemented using the *varSelRF* package [83] to eliminate the least important input variables based on 100,000 trees in the first forest and 50,000 trees in all subsequent iterations. Ten replicates of each analysis were performed, and miRNA inputs appearing in ≥3 outputs are reported. MDS plots were generated to visualize group clustering based on minimum input variable sets identified by Random Forests.

### Gain and loss evolution

To investigate miRNA evolution, we used our new Weddell seal annotation, the miRNA annotations of five domestic mammals presented in [33], as well as the available miRBase annotations for human and mouse. All hairpin sequences were clustered using *CD-hit* [84] with 80% minimum identity. Following the method described in [33], we identified syntenic regions between pairs of genomes, thus recovering conserved microRNA loci which had been missed in one or more species (likely due to a lack of sequencing depth). In order to do so, all miRNA loci annotated in each species were aligned, using BLASTN [85], against the latest genome assemblies of the remaining 7 species [33]. BLAST hits to each assembly were filtered for an e-value ≤ 10^− 6^ and an alignment length of at least 40 nucleotides. These hits were treated as putative homologous miRNA loci. We then identified the closest protein coding gene upstream and downstream of the selected hits, as well as the gene containing the hit for all intragenic hits. Genes surrounding or containing the query (for example, a Weddell seal miRNA) and the subject sequences (for example, a BLAST hit in the canine genome) were compared, checking for the presence of at least one homologous pair of genes with conserved synteny structure (i.e. same upstream or downstream gene, both on the same or on the opposite strand with respect to the miRNA/BLAST hit). Any BLAST hit supported by synteny conservation of at least one protein coding gene was then considered an orthologous miRNA locus. The presence absence matrix of orthogroups across all 8 species was then updated, considering these synteny-supported orthologous loci as miRNA genes. We then used *Dollo* parsimony to infer the evolutionary patterns of gain and loss for each orthogroup across the phylogenetic tree. The patterns of gain and loss of miRNA clusters across the phylogeny was estimated using *dollop* from the package *phylip-3.696* [86].

### Differential expression analyses

We used *DeSeq2* [81] to identify miRNAs that were differentially expressed (DE) among tissues or between developmental stages (pup versus adult). Developmental stage was input as a fixed factor for the tissue comparison analysis. We adopted a conservative approach to identify tissue-specific DE miRNAs, identifying only the most highly (or lowly) expressed miRNAs by requiring that a given miRNA be similarly up- or down-regulated in all pairwise comparisons with the other tissues (i.e. a miRNA was considered up-regulated in brain if it was higher in brain compared to each of heart, muscle, and plasma). Moreover, for the DE miRNA to be considered in downstream analyses, we required the total number of mapped reads across biological replicates to be 30 or more in at least one triplet of biological replicates (e.g., a total of 30 reads across the 3 pup brain samples). This requirement filtered 11 upregulated miRNA strands (3 in brain, 1 in muscle and 7 in plasma) from further analysis. DE miRNA are presented as adult state relative to pup (i.e. an upregulated miRNA is higher in adults), and were evaluated first a single pairwise comparison, with tissue included as a fixed factor, and then in each tissue alone. Among the list of DE miRNAs between pup and adult in each tissue, one miRNA strand (downregulated in brain) was filtered based on the minimum read count criterion. *DeSeq2* results were corrected for false discovery rate using the method of Benjamini-Hochberg [87], and miRNAs were considered differentially expressed if FDR-corrected q value ≤ 0.05.

### Target predictions, gene ontology, and pathway analyses

We predicted miRNA target sites by comparing miRNA sequences (for 3p and 5p forms) from our new annotation against the UTR sequence alignments from 72 vertebrates available from *TargetScan7* [68] (7mer and 8mer interactions with a *weighted context++ score* ≤ − 0.1), *miRanda* [88] (score > 140), and *Pita* [89] (7mer and 8mer interactions with no mismatches and a score ≤ − 10). Only miRNA:target interactions (i.e. a specific miRNA targeting a particular 3’UTR site) predicted by all 3 tools were retained for gene enrichment analysis.

Gene ontology (GO) analyses were performed using the R package *gProfileR* [90] or with DAVID Functional Annotation [91, 92]. We performed GO analysis on each set of mRNA targets for DE miRNAs, using FDR *p*-value correction, compared to a custom background, represented by the complete (non-redundant) list of genes used for target prediction analysis. This was obtained by mapping the list of human Ensembl transcript ids (ENST) used for the target prediction analyses to the corresponding gene id (ENSG) using gene tables available from *TargetScan7 (http://www.targetscan.org/vert_72/)*. To evaluate enriched pathways on all targets of DE miRNAs in a single category (e.g. upregulated in brain) we used DAVID (default settings, *p* < 0.05) and applied the default human background. Significantly enriched pathways were identified by either KEGG classifiers with human disease categories removed or GO Biological Processes.

## Supplementary information

**Additional file 1: Figure S1.** Read statistics considering different stages of the bioinformatic analysis: number of raw reads; reads after adapter trimming (min. 16nt long); trimmed reads with a perfect match to the genome; trimmed reads unmapped to the genome identified as a miRBase mature miRNA sequence. **Figure S2.** Percentage of genome matching reads (no gap or mismatch) compared to the percentage of unmapped reads having a perfect match to a miRBase annotated miRNA. **Figure S3.** Venn diagram showing the intersection between miRCat2 and miRDeep2 predictions. **Figure S4.** Abundance plots for a selection of DE miRNAs. Abundance (y axis) along the hairpin sequence (x axis) is defined as reads per million genome matching. Colors separate tissues, while fill and dotted lines denote pup and adult individuals, respectively. **Figure S5.** Venn diagrams showing the overlap between sets of DE miRNAs for each comparison: “Brain”, “Heart”, “Muscle”, “Plasma” and “Adult vs. Pup”. **Figure S6.** Selected GO accessions enriched in the targets of miR-339-3p. The color gradient reflects the calculated q-value; different GO accessions are listed along the x-axis, while the fold change enrichment is shown on the y-axis. **Figure S7.** Venn diagrams showing the overlap between sets of DE miRNAs for each tissue-specific developmental comparison: “age_brain”, “age_heart” and “age_muscle”. No DE miRs were found in plasma.

**Additional file 2: Table S1.** small RNA reads aligned against the final set of 559 high confidence miRNA loci. For each locus, the full hairpin sequence is shown, followed by the set of reads (one per line) perfectly matching the locus (with the corresponding abundance) and the predictive secondary structure of the miRNA hairpin.

**Additional file 3: Table S2.** Overview of all samples used in this study. **Table S3.** number of reads mapped to each miRNA locus across all samples. **Table S4.** number of reads mapped to each miRNA locus across all samples. **Table S5.** Tissue-specific differential expression statistics considering all pairwise tissue comparisons (brain_heart, brain_muscle, brain_plasma, heart_muscle, heart_plasma, muscle_plasma). Fold change refers to changes from tissue_1 to tissue_2, as defined by column H. **Table S6.** Age-specific differential expression statistics considering all tissues together. Fold change refers to changes from pup to adult (i.e. positive sign indicates upregulation in adult). **Table S7.** Tissue-specific differential expression statistics for developmental stage (adult versus pup). **Table S8.** Significant pathway enrichments in brain, heart, and muscle for mRNA targets of all microRNAs differentially expressed in Weddell seal maturation. **Table S9.** Enriched KEGG pathways associated with mRNA targets of novel miRNAs upregulated (+) or downregulated (−) in four Weddell seal sample types. **Table S10.** Number of significant pathway enrichments annotated to mRNA targets of novel Weddell seal microRNAs that were elevated (+) or decreased (−) in four sample types.

## Data Availability

All sequencing data used in this study have been submitted to the Sequence Read Archive (https://www.ncbi.nlm.nih.gov/sra) under accession PRJNA542051.

## Abbreviations

miRNA: microRNA
FDR: False Discovery Rate
ENST: Ensembl transcript ID
ENSG: Ensembl gene ID
DE: Differential expression/Differentially expressed
