# Supplementary material for: microRNA profiling in the Weddell seal suggests novel regulatory mechanisms contributing to diving adaptation
Source: BMC Genomics. 2020 Apr 15;21:303. doi: 10.1186/s12864-020-6675-0 (PMC7158035; doi:10.1186/s12864-020-6675-0)
Supplement: Supplementary file 1 — Additional file 1: Figure S1. Read statistics considering different stages of the bioinformatic analysis: number of raw reads; reads after adapter trimming (min. 16nt long); trimmed reads with a perfect match to the genome; trimmed reads unmapped to the genome identified as a miRBase mature miRNA sequence. Figure S2. Percentage of genome matching reads (no gap or mismatch) compared to the percentage of unmapped reads having a perfect match to a miRBase annotated miRNA. Figure S3. Venn diagram showing the intersection between miRCat2 and miRDeep2 predictions. Figure S4. Abundance plots for a selection of DE miRNAs. Abundance (y axis) along the hairpin sequence (x axis) is defined as reads per million genome matching. Colors separate tissues, while fill and dotted lines denote pup and adult individuals, respectively. Figure S5. Venn diagrams showing the overlap between sets of DE miRNAs for each comparison: “Brain”, “Heart”, “Muscle”, “Plasma” and “Adult vs. Pup”. Figure S6. Selected GO accessions enriched in the targets of miR-339-3p. The color gradient reflects the calculated q-value; different GO accessions are listed along the x-axis, while the fold change enrichment is shown on the y-axis. Figure S7. Venn diagrams showing the overlap between sets of DE miRNAs for each tissue-specific developmental comparison: “age_brain”, “age_heart” and “age_muscle”. No DE miRs were found in plasma. [file 12864_2020_6675_MOESM1_ESM.pdf]

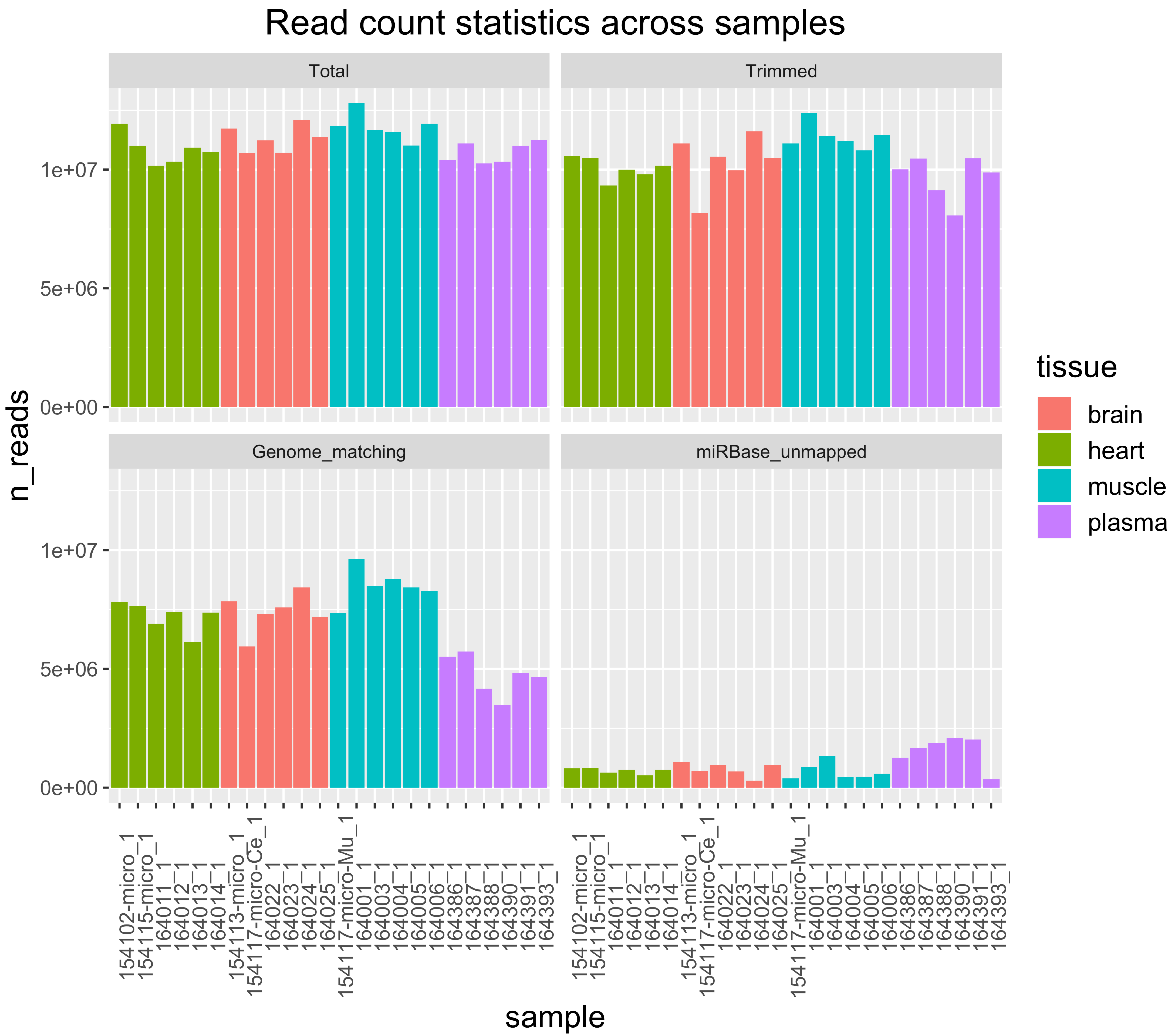

Supplementary Figure S1. Read statistics considering different stages of the bioinformatic analysis: number of raw reads; reads after adapter trimming (min. 16nt long); trimmed reads with a perfect match to the genome; trimmed reads unmapped to the genome identified as a miRBase mature miRNA sequence.

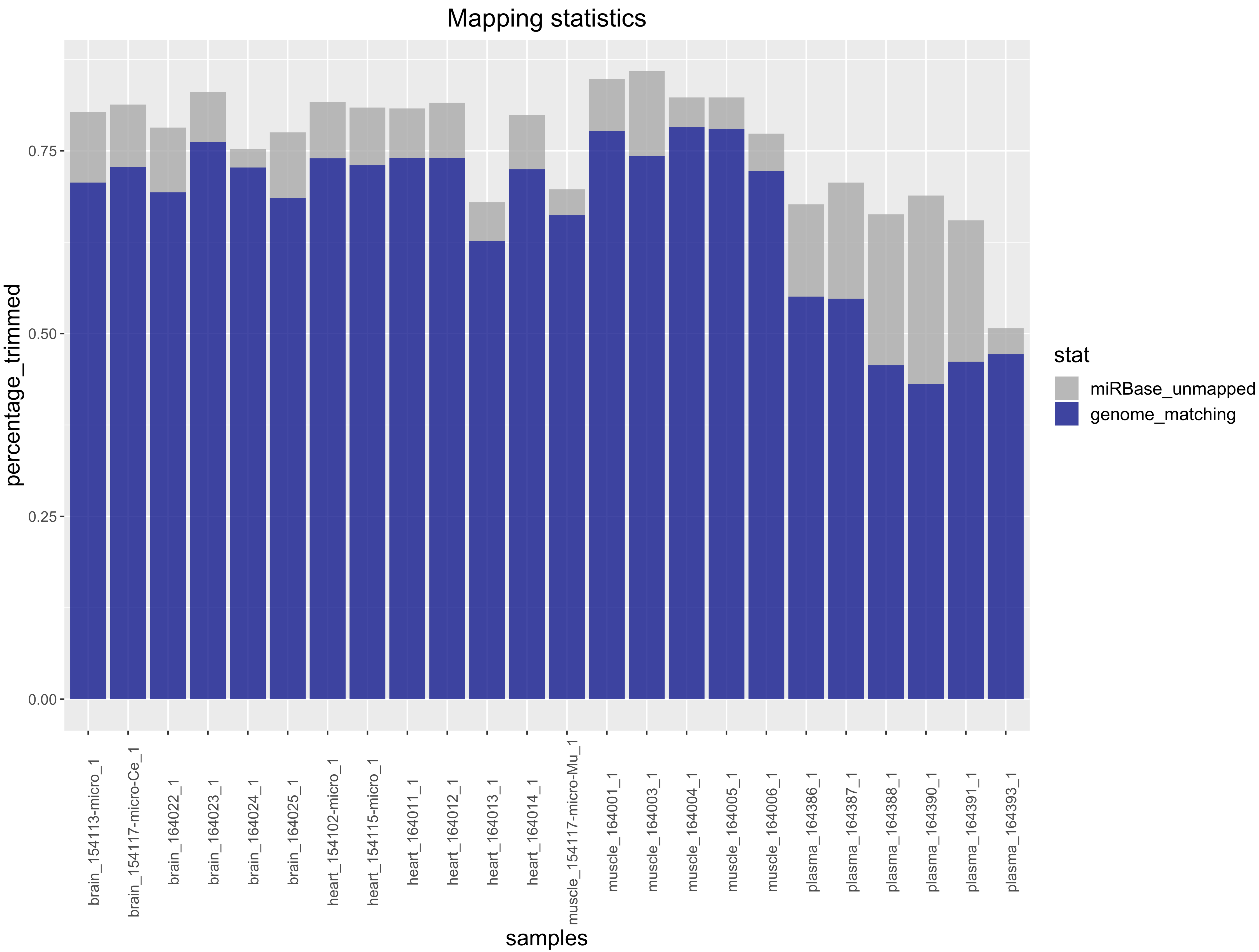

Supplementary Figure S2. Percentage of genome matching reads (no gap or mismatch) compared to the percentage of unmapped reads having a perfect match to a miRBase annotated miRNA.

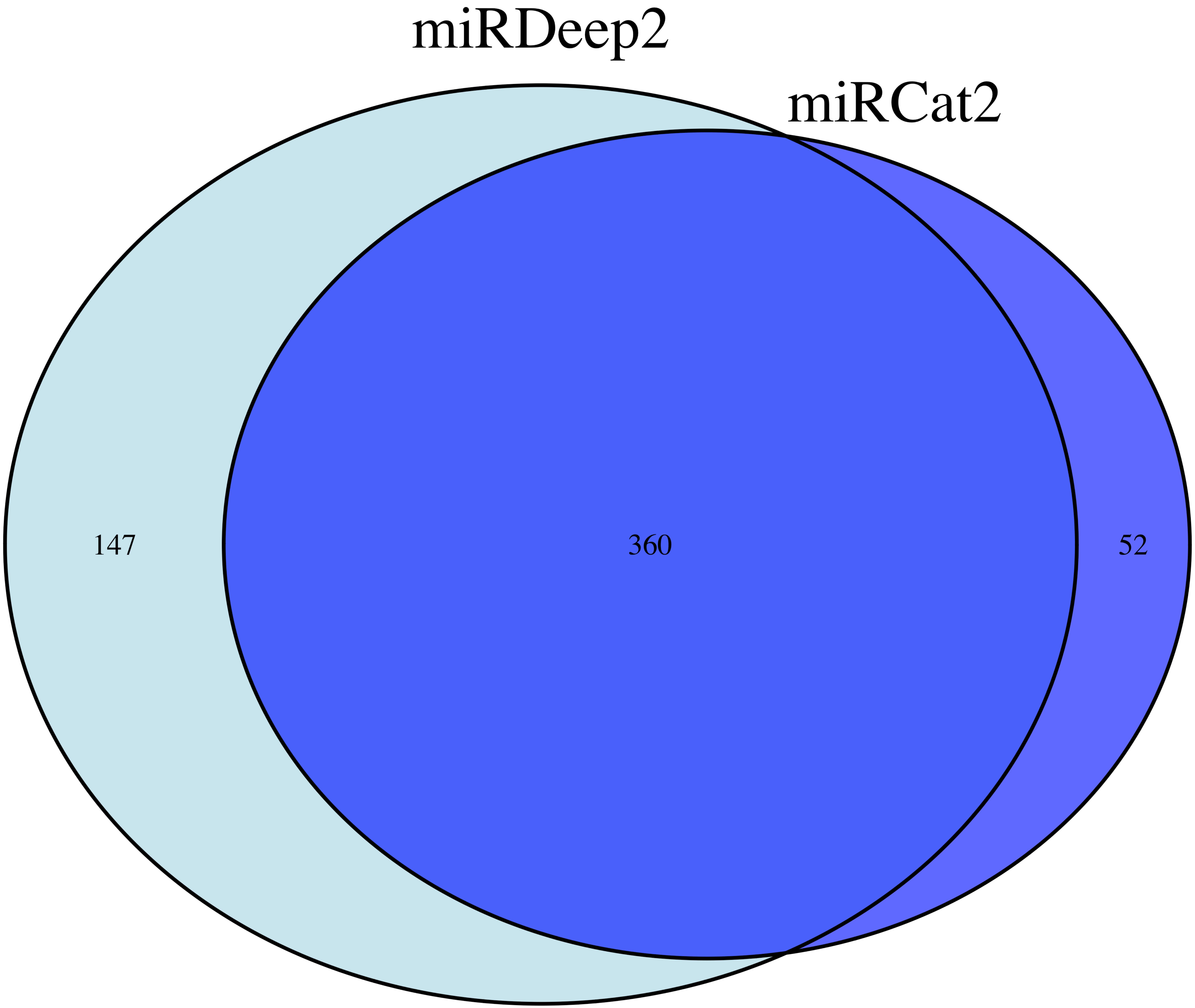

Supplementary Figure S3. Venn diagram showing the intersection between miRCat2 and miRDeep2 predictions

Supplementary Figure S4. Abundance plots for a selection of DE miRNAs. Abundance (y axis) along the hairpin sequence (x axis) is defined as reads per million genome matching. Colors separate tissues, while fill and dotted lines denote pup and adult individuals, respectively.

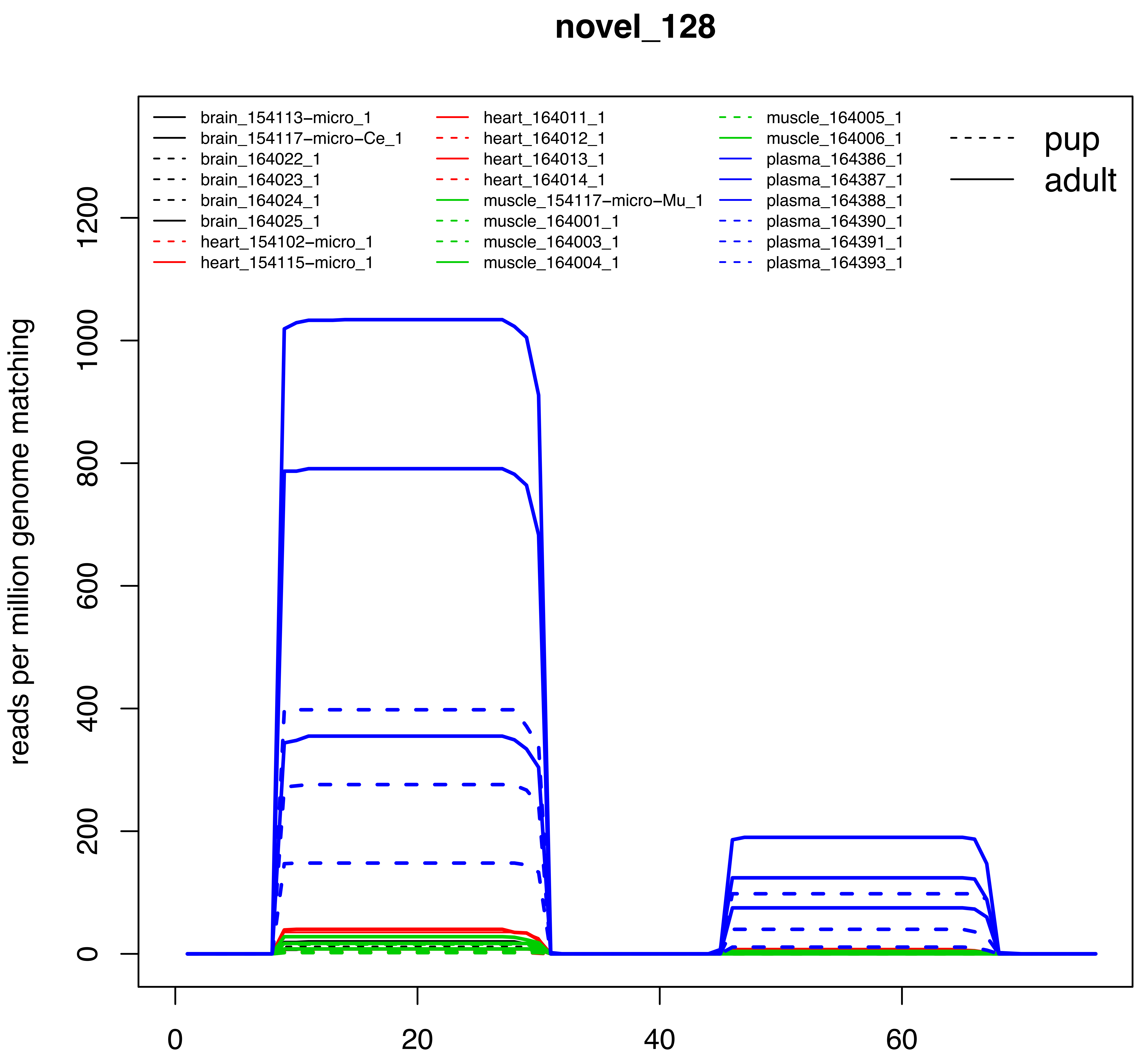

miRNA-5p: ↑ age ↓ brain

miRNA-3p: ↑ brain, plasma

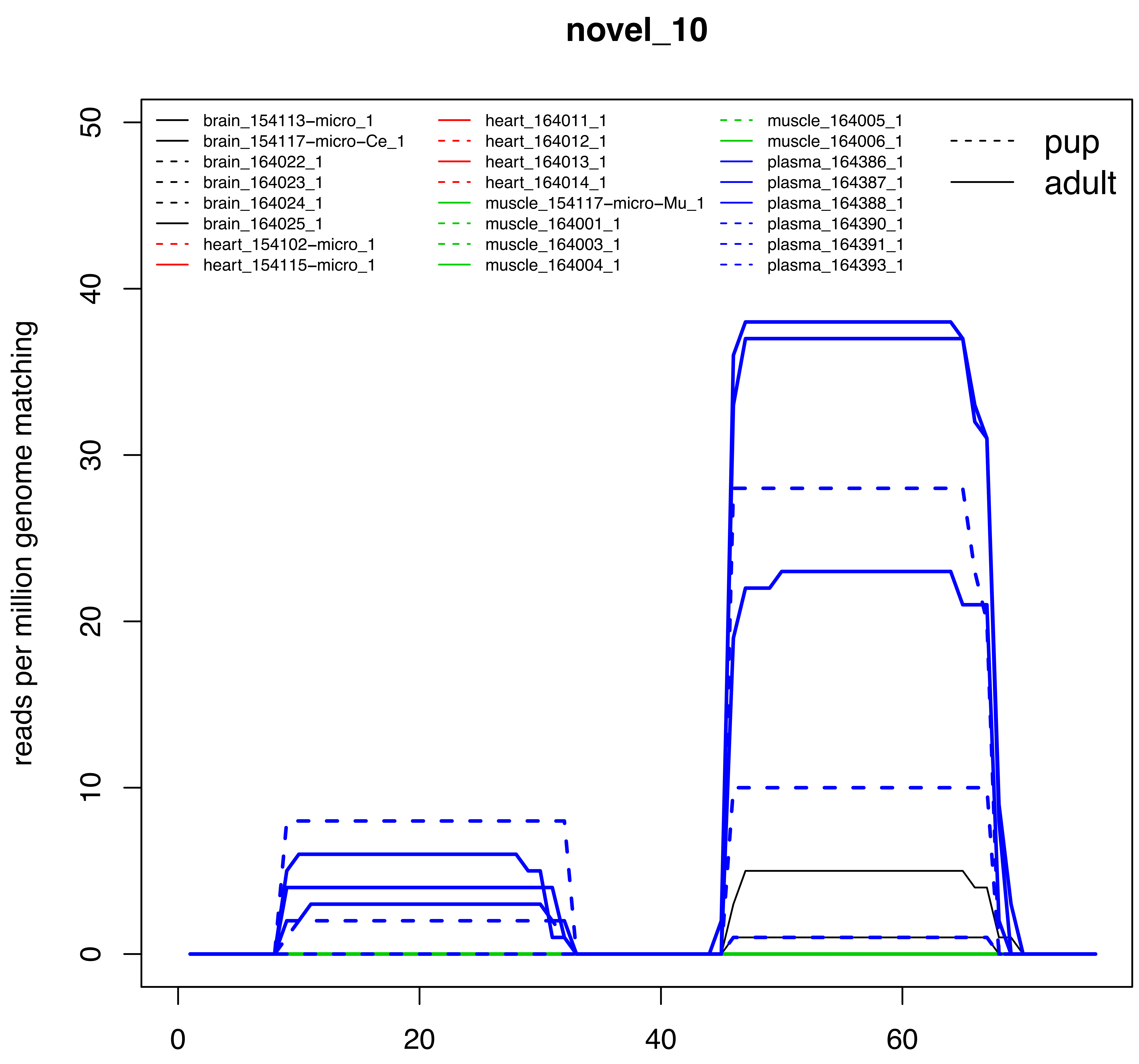

miRNA-5p: ⬆ plasma miRNA-3p: ⬆ plasma

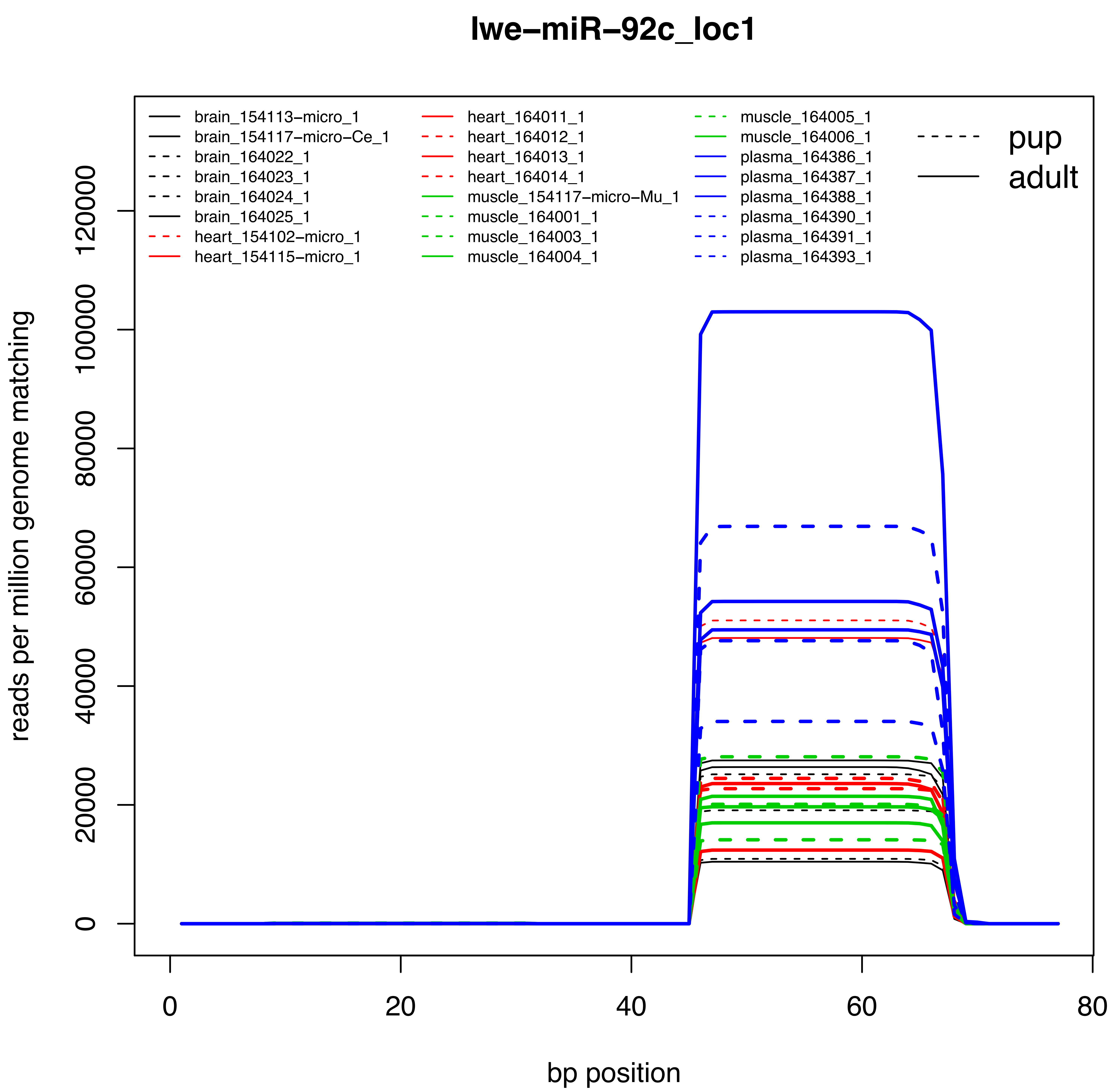

miRNA-3p: ⬆ brain, plasma

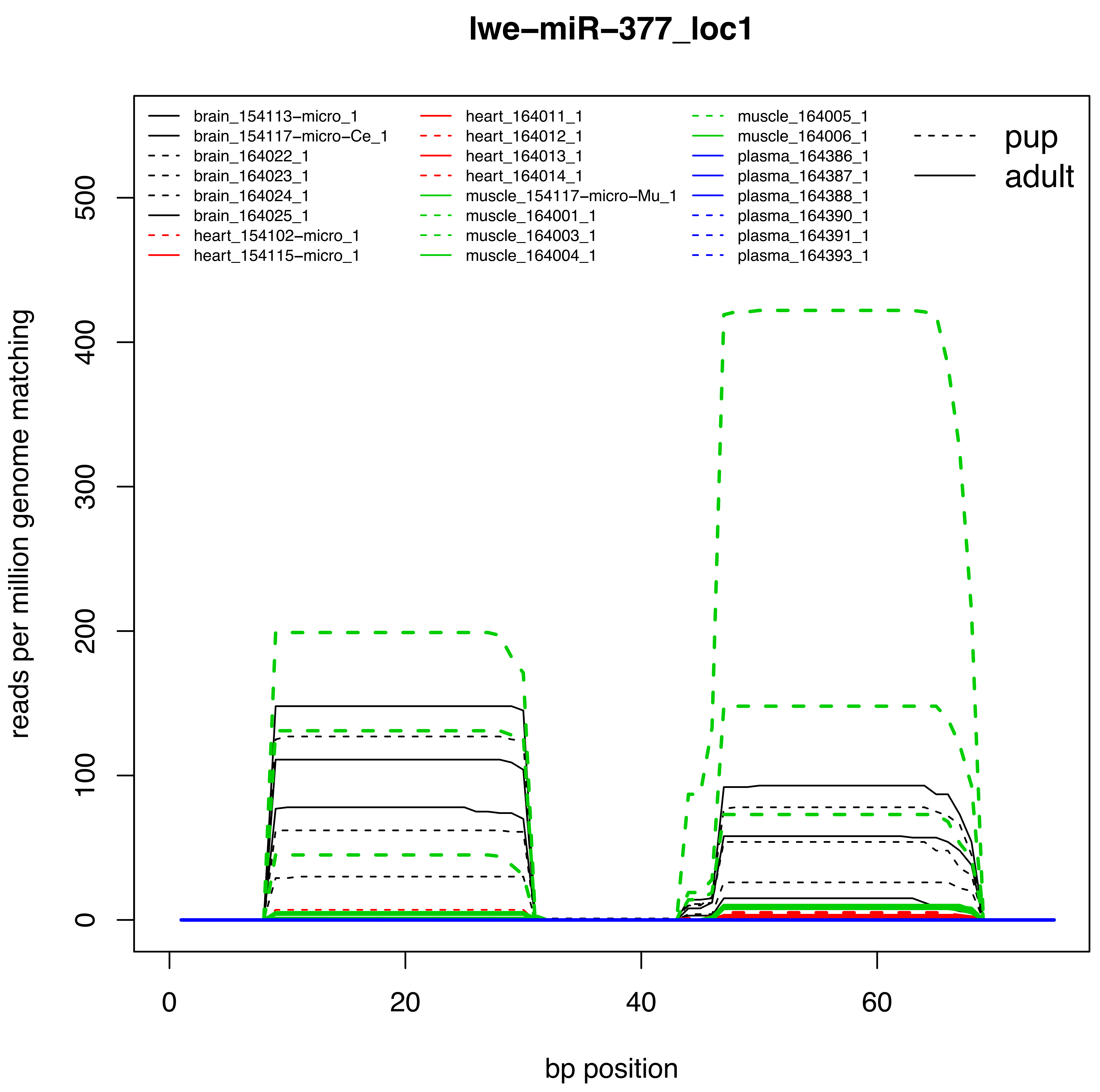

miRNA-5p: ↑ age (muscle) miRNA-3p: ↑ age (muscle)

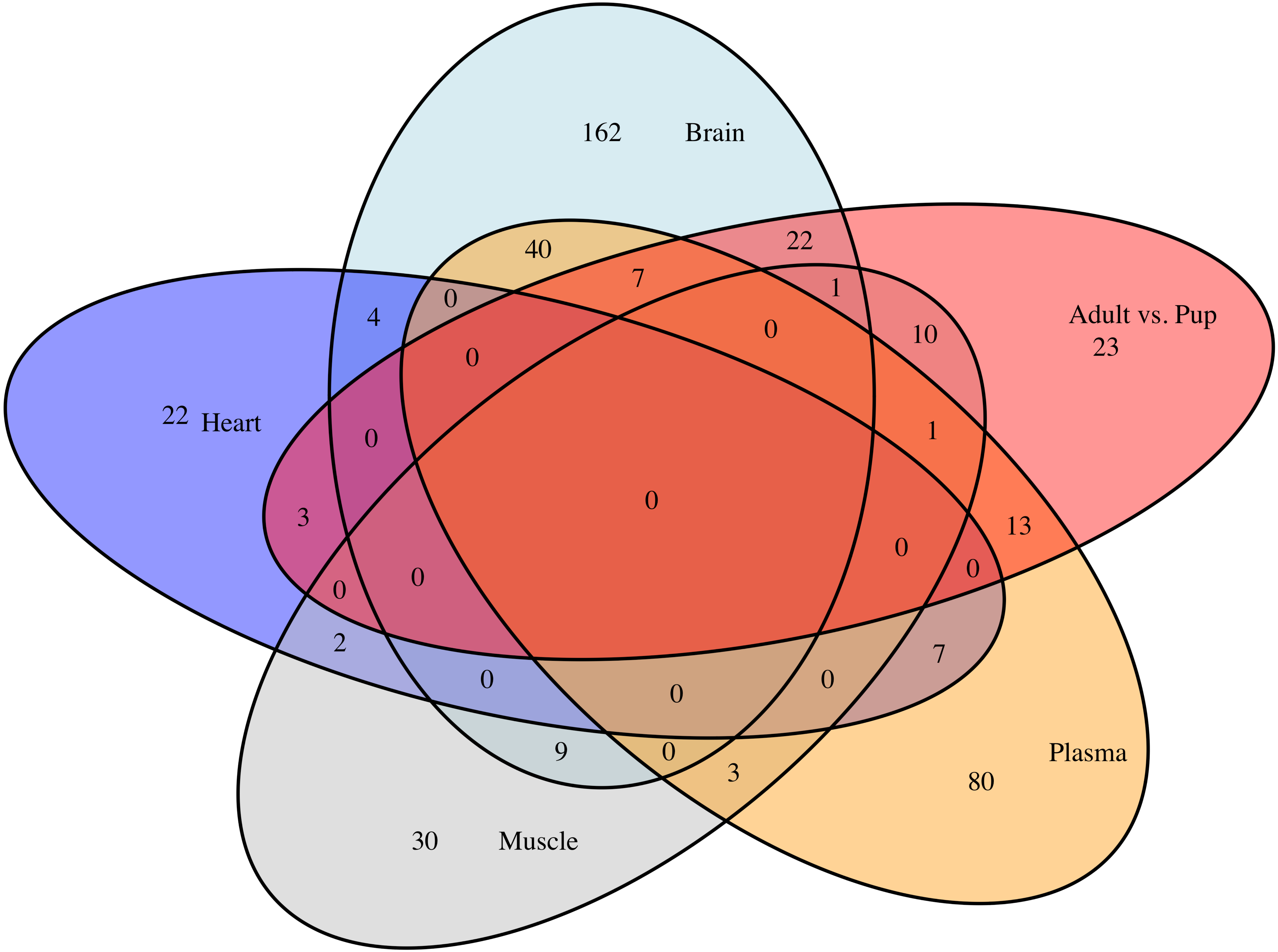

Supplementary Figure S5. Venn diagrams showing the overlap between sets of DE miRNAs for each comparison: “Brain”, “Heart”, “Muscle”, “Plasma” and “Adult vs. Pup”.

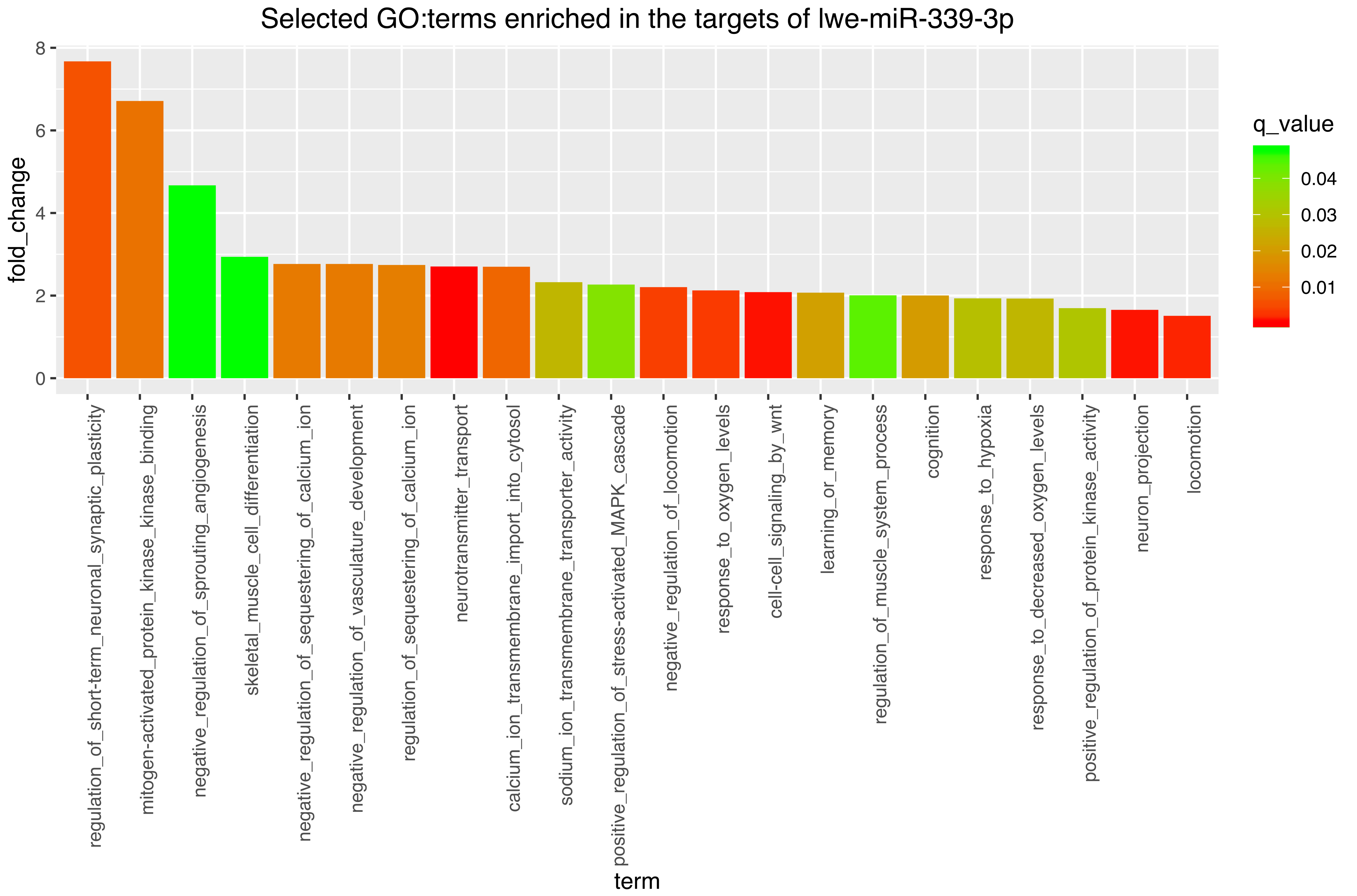

Supplementary Figure S6. Selected GO accessions enriched in the targets of miR-339-3p. The color gradient reflects the calculated q-value; different GO accessions are listed along the x-axis, while the fold change enrichment is shown on the y-axis.

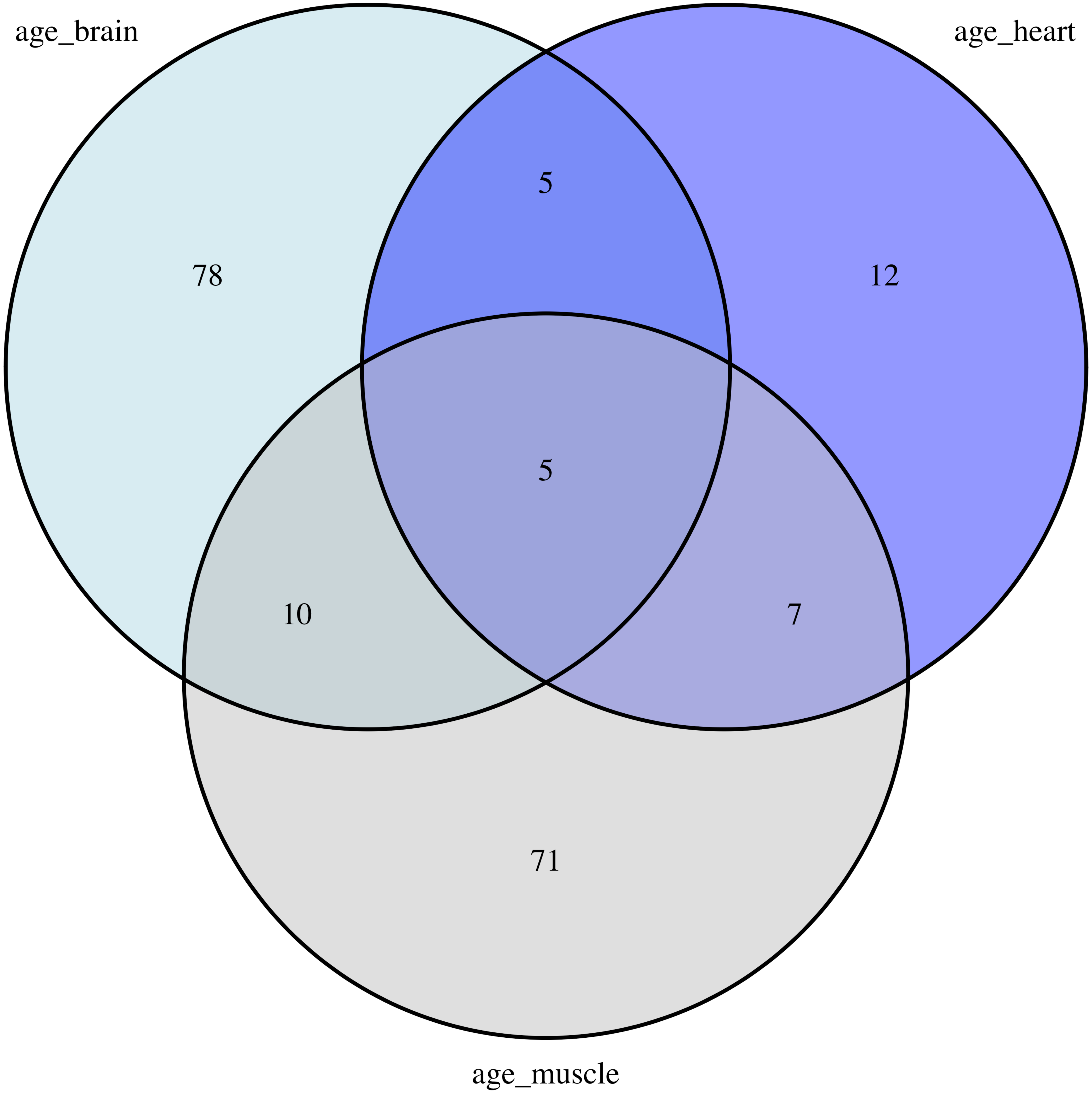

Supplementary Figure S7. Venn diagrams showing the overlap between sets of DE miRNAs for each tissue specific developmental comparison: “age_brain”, “age_heart” and “age_muscle”. No DE miRs were found in plasma.
